# Bioengineering the Oxygen-Deprived Tumor Microenvironment Within a Three-Dimensional Platform for Studying Tumor-Immune Interactions

**DOI:** 10.3389/fbioe.2020.01040

**Published:** 2020-09-04

**Authors:** Somshuvra Bhattacharya, Kristin Calar, Claire Evans, Mark Petrasko, Pilar de la Puente

**Affiliations:** ^1^Cancer Biology and Immunotherapies Group, Sanford Research, Sioux Falls, SD, United States; ^2^Histology and Imaging Core, Sanford Research, Sioux Falls, SD, United States; ^3^Sanford PROMISE, Sanford Research, Sioux Falls, SD, United States; ^4^Department of Surgery, University of South Dakota Sanford School of Medicine, Sioux Falls, SD, United States; ^5^Flow Cytometry Core, Sanford Research, Sioux Falls, SD, United States

**Keywords:** oxygen availability, three-dimensional, immune evasion, bioengineering, tumor microenvironment, breast cancer

## Abstract

Oxygen deprivation within tumors is one of the most prevalent causes of resilient cancer cell survival and increased immune evasion in breast cancer (BCa). Current *in vitro* models do not adequately mimic physiological oxygen levels relevant to breast tissue and its tumor-immune interactions. In this study, we propose an approach to engineer a three-dimensional (3D) model (named 3D engineered oxygen, 3D-O) that supports the growth of BCa cells and generates physio- and pathophysiological oxygen levels to understand the role of oxygen availability in tumor-immune interactions. BCa cells (MDA-MB-231 and MCF-7) were embedded into plasma-derived 3D-O scaffolds that reflected physio- and pathophysiological oxygen levels relevant to the healthy and cancerous breast tissue. BCa cells grown within 3D-O scaffolds were analyzed by flow cytometry, confocal imaging, immunohistochemistry/immunofluorescence for cell proliferation, extracellular matrix protein expression, and alterations in immune evasive outcomes. Exosome secretion from 3D-O scaffolds were evaluated using the NanoSight particle analyzer. Peripheral blood mononuclear cells were incorporated on the top of 3D-O scaffolds and the difference in tumor-infiltrating capabilities as a result of different oxygen content were assessed by flow cytometry and confocal imaging. Lastly, hypoxia and Programmed death-ligand 1 (PD-L1) inhibition were validated as targets to sensitize BCa cells in order to overcome immune evasion. Low oxygen-induced adaptations within 3D-O scaffolds validated known tumor hypoxia characteristics such as reduced BCa cell proliferation, increased extracellular matrix protein expression, increased extracellular vesicle secretion and enhanced immune surface marker expression on BCa cells. We further demonstrated that low oxygen in 3D-O scaffolds significantly influence immune infiltration. CD8+ T cell infiltration was impaired under pathophysiological oxygen levels and we were also able to establish that hypoxia and PD-L1 inhibition re-sensitized BCa cells to cytotoxic CD8+ T cells. Bioengineering the oxygen-deprived BCa tumor microenvironment in our engineered 3D-O physiological and tumorous scaffolds supported known intra-tumoral hypoxia characteristics allowing the study of the role of oxygen availability in tumor-immune interactions. The 3D-O model could serve as a promising platform for the evaluation of immunological events and as a drug-screening platform tool to overcome hypoxia-driven immune evasion.

## Introduction

With 1 million new cases in the world every year, breast cancer (BCa), excluding skin cancer, is the most common cancer in women (Bray et al., 2018). Close to 1 in 8 women will be diagnosed with BCa in their lifetime, accounting for almost 18% of all cancer in women (Sun et al., 2017). As of 2018, the American Cancer Society estimates that each year about 2,000 new cases of invasive BCa are being diagnosed (Siegel et al., 2019). Despite breast tumors being more manageable now, mortality from BCa resistance, recurrence and metastasis is still the number one challenge that BCa therapy has consistently failed to eliminate (Vondeling et al., 2018). Cancer immune surveillance consists of the ability of the immune system to recognize and eliminate tumor cells (Swann and Smyth, 2007). BCa tumor cells avoid tumor healing processes intrinsically executed by the patient’s host immunity hence undergoing sustained survival (Domschke et al., 2016). While a demonstrated durable response to immunotherapeutic intervention has been shown in some types of cancer, such as melanoma, bladder, and renal cell carcinoma, BCa has not shown the same level of efficacy (Bates et al., 2018).

In regard to this, many studies have been directed toward understanding the mechanisms of immune evasion in BCa (Kogan et al., 2018). The knowledge gained from some of these studies has generated many efficient immune-therapeutic targets, which function either by boosting a patient’s intrinsic immune arsenal, e.g., immune checkpoint inhibitions (Taylor et al., 2017) or recovering immune operational performance by reducing the influence of immune-suppressive molecules generated by the BCa tumor (Hanna et al., 2019). Increasing evidence suggests that regulation of immune evasion in BCa extends well beyond the regulation of just the resident tumor cells. BCa tumor microenvironment (TME) parameters such as extracellular matrix (ECM), cell-matrix interactions, growth factors, cytokines, and an oxygen-deprived niche can heavily contribute to immune evasion as well (Vannini et al., 2019).

Intratumoral hypoxia, defined by the deprivation of oxygen within tumors, has been associated with modified cellular expression levels, altered ECM secretion, and active immune evasion (Jarman et al., 2019). Patients with poorly oxygenated tumors have an increased risk of mortality (Walsh et al., 2014). Also, the presence of tumor-infiltrated lymphocytes (TILs) can be used as a relevant prognostic indicator (Fu et al., 2019). A notorious hallmark associated with BCa intratumoral oxygen deprivation is an alteration in the cancer cell survival metabolism to function through the hypoxia-inducible factor-1 alpha (HIF-1α) regulatory pathway, which is an essential mediator of tumor response to hypoxia that includes the resistance of tumor cells to apoptosis (Peir et al., 2019). Physiological oxygen content can vary widely from tissue to tissue. While healthy breast reportedly comprises an average oxygen partial pressure (pO_2_) content of 8.6 kPa (65 mm Hg) to 5.9 kPa (45 mm Hg), oxygen deprivation within the BCa tumor can take this content down to 1.3 kPa (10 mm Hg) and in about 30% of these breast tumors to less than 0.2 kPa (2 mm Hg) (Hohenberger et al., 1998; Williams et al., 2001; Vaupel et al., 2007; Rundqvist and Johnson, 2013; McKeown, 2014).

In the efforts to recreate oxygen deprivation-driven tumor immune evasion in the laboratory, very few cancer *in vitro* models adequately mimic physiological oxygen levels relevant to breast tissue and its tumor-immune interactions. Traditional two-dimensional (2D) culture models fail to generate physiologically relevant oxygen contents, and hence experiments using these models expose the cells to higher than physiological oxygen levels (Ast and Vamsi, 2019). These models might not accurately demonstrate tumor-immune evasion. To overcome these limitations, three-dimensional (3D) culture models have been utilized. A wide array of matrices, including synthetic and natural, have been developed to recapitulate critical features of the TME (Padhye et al., 2019). While biochemical and physical parameters, such as conduciveness to vital biochemical signals, stiffness, degradability, permeability to nutrients, diffusibility to gases and swelling indices have been heavily studied (Sahoo et al., 2005; Grimes et al., 2014a,b; Hao et al., 2016; Rijal and Li, 2018; Vega et al., 2018; Wullkopf et al., 2018), how tumor-immune interactions can be modulated within an oxygen deficient microenvironment remains under-investigated.

Therefore, the purpose of our study is to understand the role of oxygen availability in tumor-immune interactions. In this regard, we bioengineered an *in vitro* model, 3D engineered oxygen (3D-O) that supports the growth of BCa cells, generates physio- and pathophysiological breast oxygen levels, and exhibits hypoxia-driven BCa tumor-immune evasive outcomes. We hypothesize that the results obtained from the 3D-O model can help understand oxygen-specific adaptations within the tumor and hence help to further investigate the prevailing low oxygen-driven consequences in tumor-immune interactions.

## Materials and Methods

### Reagents

Calcium chloride (CaCl_2_), trans-4-(Aminomethyl) cyclohexanecarboxylicacid (AMCHA), dimethyl sulfoxide (DMSO), Ficoll-Paque density gradient medium, DAPI, and glutaraldehyde, were purchased from Sigma-Aldrich (Saint Louis, MO). Type I collagenase and Image-iT^TM^ Green Hypoxia detection reagent and Triton X-100 were purchased from Thermo Fischer Scientific (Waltham, MA). Cell tracker DiO (excitation, 488 nm; emission, 525/50 nm) was purchased from Invitrogen (Carlsbad, CA). Drugs including PX-478 and Durvalumab were purchased from Selleck Chemicals (Houston, TX).

### Cell Lines

The BCa cell lines representing different molecular subtypes (MDA-MB-231: Triple negative and MCF-7: Luminal A) used in this study were kind gifts from Dr. Kristi Egland (Sanford Research, Sioux Falls, SD). All human cell lines used in this study were authenticated by short tandem repeat profiling (Genetica DNA Laboratories, Cincinnati, OH). Also, all cell lines were confirmed mycoplasma free. Cell lines were cultured at 37°C, 5% CO_2_ in DMEM media (Corning CellGro, Mediatech, Manassas, VA) which was supplemented with 10% fetal bovine serum (FBS, GiBCo, Life technologies, Grand Island, NY), 100 U/ml penicillin, and 100 μg/ml streptomycin (Corning CellGro, VA). Before experiments, in some cases, BCa cells (1 × 10^6^ cells/ml) were prelabeled with DiO (10 μg/ml) for 1 h.

### Primary Cells

Primary peripheral blood mononuclear cells (PBMCs) were isolated from healthy blood provided by the Sanford USD Medical Center, Sioux Falls, SD using SepMate PBMC isolation tubes (STEMCELL Technologies, Canada) following manufacturer instructions. Informed consent was obtained from all healthy subjects with approval from the Sanford Health Institutional Review Board and in accordance with the Declaration of Helsinki. Collected fresh blood was first centrifuged at 3000 rpm for 10 min at room temperature, to separate plasma. After plasma isolation, leftover blood was then diluted with phosphate-buffered saline (PBS) containing 2% FBS in equal volume to blood and pipetted slowly along the wall of the SepMate tube in which Ficoll-Paque density gradient medium was added beforehand. After 10 min of centrifugation with the brake on, highly purified PBMCs were poured into a new tube. Finally, the cells were washed three times by centrifuging at 100 × *g* for 10 min at room temperature and suspending in PBS containing 2% FBS solution for each wash cycle. The enriched cells were counted and cells were viably frozen at −80°C in FBS with 10% (v/v) DMSO. PBMCs were pre-analyzed by flow cytometry to determine the frequency of the immune cell population before using them in any experiment to account for variability between patients.

### Development of Plasma-Derived 3D Matrices

**FIGURE 1:**
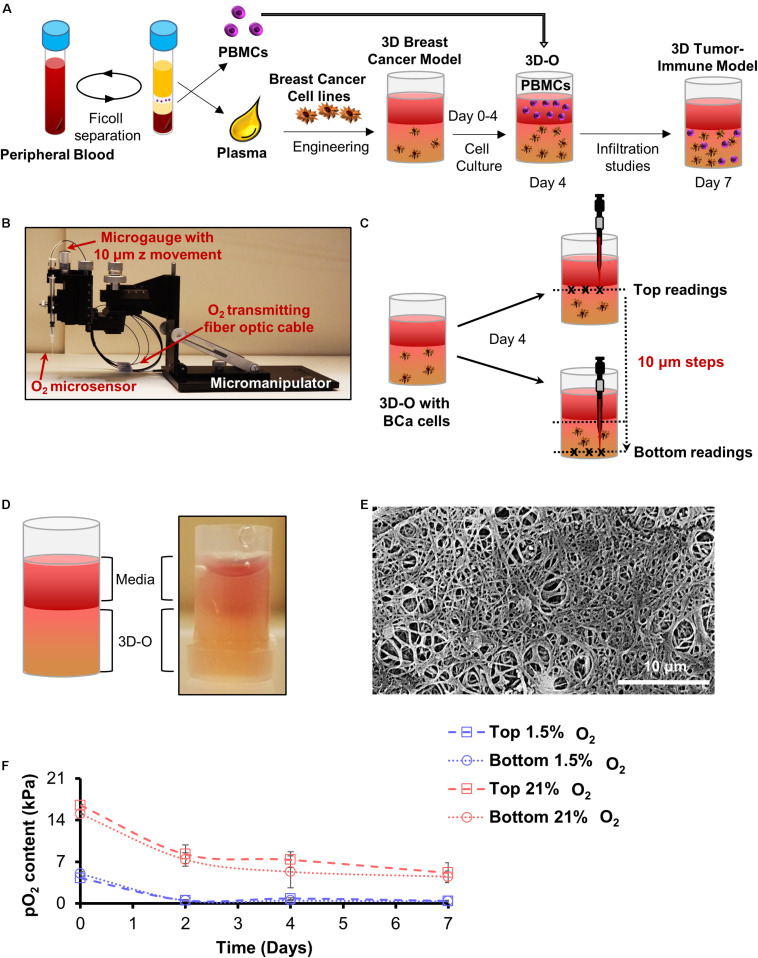
Development of 3D-O matrices. **(A)** 3D-O matrices were developed through cross-linking of fibrinogen (naturally found in the plasma of blood supernatant) including BCa cells. PBMCs were added on top of the 3D-O scaffolds on day 4 of culture and allowed to infiltrate into the scaffold until day 7. **(B)** Oxygen microsensor and Manual Micromanipulator configuration for O_2_ profiling in the Z-direction every 10 mm. **(C)** Oxygen microsensor was extended delicately and safely with 10 mm profiling accuracy to determine surface (border between media and 3D-O matrix) and bottom (bottom of the well) readings. **(D)** Visual and schematic representation of a 3D-O matrix with the medium on top. **(E)** SEM micrograph of acellular 3D-O scaffold cultured on day 4. Scale bar: 10 μm. **(F)** Average top to bottom pO_2_ levels (kPa) obtained from 3D-O matrices embedded with MDA-MB-231 and MCF-7 BCa cells, incubated up to 7 days under 21% and 1.5% O_2_.

Using methods previously described (de la Puente et al., 2011, 2015; de la Puente and Ludena, 2014), a biological matrix was engineered through the cross-linking of plasma fibrinogen to fibrin. Calcium chloride (CaCl_2_) and AMCHA were used as a cross-linker and stabilizer, respectively. A detailed description of the methodology and crosslinking optimization for the 3D model utilized in this study has been elaborately discussed in a previous study (Calar et al., 2020). Briefly, to prepare a plasma-derived 3D matrix, a mixture of plasma, BCa cell suspension (at a concentration of 0.3 million cells/ml) in DMEM complete media, AMCHA and CaCl_2_ was prepared with a 4:4:1:1 volume ratio, respectively. To avoid intra-assay variability due to the matrix, plasma was pooled in batches. Matrices were made in 96 well plates (gel volume 100 μl and gel height was approximately 3 mm). Matrices were allowed to stabilize in an incubator at 37°C for 2.5 to 3 h, before being covered with DMEM complete media. After gelification was completed, plasma-derived 3D matrices were incubated for 4 days at 37°C, while being exposed to variable O_2_ environments (21% and 1.5% O_2_). The novelty of the model in this study is the manipulation of oxygen levels within the matrix and how these matrices can efficiently mimic the niche corresponding to variable oxygen levels as seen in the breast TME. Plasma-derived 3D matrices recapitulating physio- and pathophysiological oxygen levels from here on referred to as 3D engineered oxygen (3D-O) matrices were used as a tool to study BCa behavior and tumor-immune interactions with varying oxygen levels. In the case of tumor-immune infiltration assays, PBMCs were incorporated at day 4 as a cell suspension in the medium added on the top of the matrix [1:3 to 1:6 cancer: PBMCs ratio (Owens et al., 2018)] and kept at 37°C, while being exposed to the same O_2_ environment up to day 7, e.g., Figure 1A.

### Oxygen Measurement Within 3D-O Matrices

Oxygen partial pressure (pO_2_) levels were measured in BCa cell-seeded 3D-O matrices incubated under variable O_2_ environments (21% and 1.5% O_2_)after 0, 2, 4, and 7 days of culture. 3D-O scaffolds containing BCa cells were profiled along the z-direction with an oxygen microsensor (Needle-Type Oxygen Microsensor NTH-PSt7, PreSens, Regensburg, Germany) and a manual micromanipulator (Figure 1B). Oxygen measurement by this type of sensor has been described previously (Weyand et al., 2015). Briefly, to record oxygen pressure, the sensor was introduced into the geometric center (3 measure points) of the 3D-O and moved from the border between the media and 3D-O (top) in 10 μm steps toward the bottom of the well plate, as illustrated in Figure 1C. The Software, PreSens Profiling Studio, enabled the measurement of variable step sizes, measuring velocities and wait times. Before application, a two-point calibration was performed: 1.5% O_2_ in an enclosed chamber as 1.5% O_2_ reference and ambient air as 21% O_2_ reference.

### Scanning Electron Microscopy (SEM)

Samples for SEM analysis were subjected to gradual cooling (−20°C to −80°C) after fixation with 10% glutaraldehyde solution for 6–8 h. After the fixed samples were completely frozen, the samples were subjected to lyophilization for 48 h at about −105°C/100 mTorr vacuum. Freeze-dried samples were attached to an aluminum sample stub with conductive silver paint (Ted Pella, Inc., Redding, CA) and tightened using copper tape. Samples were then subjected to sputter-coating with a layer of ∼10 nm gold film before imaging by SEM. SEM images were acquired using an FEI Quanta 450 Scanning Electron Microscope at different magnifications. The accelerating voltage applied was 10 kV, and images were acquired using a dwell time of 30–60 μs.

### Immunohistochemistry (IHC) and Immunofluorescence (IF)

3D-O matrices containing BCa cells (cell-seeded) were fixed in 10% neutral buffered formalin and processed on a Leica 300 ASP tissue processor. The matrices were oriented on the slide so that the top of the matrix section always faced forward in order to allow the identification of top vs. bottom areas of the scaffolds. Paraffin-embedded matrix sections were longitudinally sliced at 10 μm. The BenchMark^®^ XT automated slide staining system (Ventana Medical Systems, Inc., AZ) was used for antibody optimization and staining. The antigen retrieval step was performed using the Ventana CC1 solution, which is a basic pH tris based buffer. Both primary and secondary antibodies were prepared in a 1X permeabilization buffer (BioLegend, CA). The Ventana iView DAB detection kit was used as the chromogen, and the slides were counterstained with hematoxylin. Anti-Ki-67 (CRM325, 1:100, Biocare Medical), anti-CD3 (MA5-12577, 1:20, Invitrogen, CA), anti-CD44 (ab157107, 1:1000, Abcam, MA) and anti-CD8 (MA5-14548, 1:100, Invitrogen, CA) primary antibodies were used. IHC images were imaged using an Aperio VERSA Bright field Fluorescence and FISH Digital Pathology Scanner (Leica, NJ). The omission of the primary antibody served as negative control. Secondary antibodies used were biotin-conjugated goat anti-Rabbit IgG (111-065-144, 1:1000, Jackson ImmunoResearch, PA) and biotin-conjugated donkey anti-mouse IgG (715-065-151, 1:1000, Jackson ImmunoResearch, PA), respectively. Acquired IHC images of 3D-O sections were analyzed using the positive pixel count algorithm in ImageScope version12.4.2.5010 (Leica, NJ). Briefly, two rectangular annotations of similar dimensions were drawn over each section in a way that the whole section was accounted for. While the first annotation box encompassed the top half of the section (top-to-middle), the second covered the bottom half of the section (middle-to-bottom). Once the annotation boxes were defined, the positive pixel algorithm was tuned to reflect accurate counting of desired positive pixels by selecting a small region of interest within each annotation box. Once the pixel identification parameters were selected, the set macro was implemented to evaluate the number of strong positive pixel counts (positive) and the number of negative pixel counts (negative) from each annotation. The macro was kept consistent between sections. Positive pixel count algorithm results reported by Imagescope were on the basis of the amount of a specific stain present in the digitized slide of interest and did not include non-stained areas. For IF studies, paraffin sections were dewaxed in the following order: 10 min in xylene, 10 min in 100% ethanol, 10 min in 95% ethanol, 10 min in 70% ethanol and 10 min in distilled water, followed by rehydration in wash buffer (0.02% BSA in PBS) for 10 min. After this, sections were subjected to incubation in blocking buffer (5% BSA in PBS) for 60 min at room temperature to block non-specific staining between the primary antibodies and the sample. Sections were rinsed with washing buffer and incubated in incubation buffer (1% BSA in PBS) with different primary antibodies. Primary antibody incubation was carried out overnight at 4°C to allow for the optimal binding of antibodies to sample targets and reduce non-specific background staining. Anti-collagen-I (MA1-26771, 1:100, Thermo Fischer Scientific, MA), anti-collagen-III (SAB4200749, 1:100, Sigma Aldrich, MO), anti-fibronectin (SAB4200784, 1:100, Sigma Aldrich, MO) and an AlexaFluor 647 conjugated anti-HIF-1α were used (359706, 1:100, BioLegend, CA). A FITC conjugated secondary antibody (SAB4600042, 1:1000, Sigma Aldrich, MO) was used whenever applicable. For samples stained with anti-HIF-1α, blocking and incubation buffers were prepared in 1X permeabilization buffer (BioLegend, CA). The dilution of antibodies was carried out according to the manufacturer’s instructions. Lastly, a drop of anti-fade mounting media containing DAPI was added to the samples and sections were imaged.

### Confocal Imaging and Analysis

3D-O matrices containing BCa cells alone or in co-culture with PBMCs growing in 8 well chambers and paraffin section cuts of 3D-O matrices were imaged using a Nikon Ti2-A1TR confocal microscope (x20 dry, x40 oil and x60 oil objectives, 2.5 magnified) and analyzed using NIS elements software (Nikon, Melville, NY, United States). Oxygen deprivation was imaged by using Image-iT^TM^ Green Hypoxia reagent (5 μM) on the day of analysis. Samples were excited at 488 nm (FITC/DiO/Image-iT^TM^ Green Hypoxia Reagent), 358 nm (DAPI), 640 nm (APC-Cy7) and the emission light was collected at 500–530 nm, 461 nm, and 650 nm long pass, for each channel, respectively. Z-stack images of approximately 0.8 mm thickness were taken for each sample at 2 μm step sizes. Each frame consisted of a 520 × 520 pixel image, taken at a rate of 1 μs/pixel. For analysis purposes, the green fluorescence from acquired images reflecting Hypoxia Image^IT^ reagent was converted to red to demonstrate the co-localization of the hypoxia stain (as re-colored in red) and DAPI (blue) of the cells. BCa cells within 3D-O physiological and 3D-O tumorous matrices were measured using various multi-step automated modules within NIS Elements (Nikon Instruments, NY) software. First, a threshold using the GFP channel (recolored now as red) was generated to map the cells of interest. The binary was created using the auto detect feature. To avoid bias, the smallest object was used to generate the binary via the auto detect feature. Values corresponding to different features of interest such as EqDiameter, Circularity, Elongation, and Mean intensity were employed to implement restrictions while recording the absolute number of cells to minimize artifacts. Additionally, to assess clustering, specific binary restrictions were utilized for each parameter that ranged between the largest observed value as the upper confidence interval and 50% higher than the calculated mean value as the lower confidence interval. The number of objects inside this defined range were selected as positive clusters. As for all the images in the z-stack, the X and Y positions were pre-defined. In order to determine extent of co-localization of stain with cell, the Pearson’s coefficient was determined using the NIS Elements software module between blue (DAPI) stained cells and green (Hypoxia Image^IT^ reagent stained cells) that were reverted to red color. For each condition, all detected cells were analyzed per experiment.

### Extracellular Vesicle Isolation and Characterization

Extracellular vesicle isolation and characterization were performed as previously described (Madeo et al., 2018) using similar protocols. Briefly, 3D-Os were seeded with MDA-MB-231 BCa cells and cultured for 4 days in DMEM medium containing 10% FBS that was depleted of exosomes. FBS was made extracellular vesicle free by performing overnight ultracentrifugation at 100,000 × *g*. 3D-O matrices were enzymatically digested with collagenase (20 mg/ml for 2–3 h at 37°C) on day 4. Sample of digested matrix components, conditioned medium samples that were collected after 4 days and extracellular vesicles were purified by differential ultracentrifugation: first, samples were centrifuged at 300 × *g* for 10 min at 4°C to pellet out the cells. Then, the supernatant was centrifuged at 2,000 × *g* for 20 min at 4°C, followed by transferring to a new tube, and then centrifugation for 30 min at 10,000 × *g*. Finally, the samples were subjected to a final spin using a SureSpin 630/17 rotor for 120 min at 100,000 × g. All pellets were washed in PBS and re-centrifuged at the same speed and re-suspended in 400 μL of sterile PBS. After this, the BCA protein assay kit (Thermo Fischer Scientific, MA) was utilized with some modifications to compensate for the low protein yield from extracellular vesicle preparations. Briefly, 5 μl of 10% Triton X-100 was added to an aliquot of 50 μl of purified 3D-O extracellular vesicles and incubated 10 min at room temperature. A 1:11 ratio of sample to working reagent was used and incubated in a 96-well plate for 1 h at 37°C. Absorbance at 562 nm was then assessed using a SpectraMax Plus 384 (Molecular Devices, CA) and protein concentration estimated from a BSA standard curve. Finally, the purified vesicles were characterized using the NanoSight particle analyzer (NanoSight, United Kingdom), following the manufacturer’s protocol.

### BCa Proliferation, Hypoxic Status and Surface Marker Expression Using Flow Cytometry

3D-O matrices were enzymatically digested with collagenase (20 mg/ml for 2–3 h at 37°C) on day 4. BCa cells were isolated and identified by gating cells with a high DiO signal (excitation, 488 nm; emission, 530/30 nm). Antibodies used to evaluate hypoxic status and surface marker expression were AlexaFluor 647 conjugated anti-HIF-1 α (359706, BioLegend, CA), APC-Cy7 conjugated anti-CD8 (344714, BioLegend, CA), PE-conjugated anti-PD-L1 (393608, BioLegend, CA), PE-conjugated anti-MUC-1 (355608, BioLegend, CA), and PerCP-Cy5 conjugated anti-CD73 (344014, BioLegend, CA). Cell viability was evaluated by using a Sytox Blue live-dead fluorescent dye (S34857, Invitrogen, CA) possessing excitation, 358 nm; emission, 461 nm or Live/Dead Blue cell stain (L34962, Thermo Fischer Scientific, MA). For all analyses, a minimum of 5,000 events were acquired using BD FACS Fortessa and FACSDiva v6.1.2 software or BD FACS Accuri and BS Accuri C6 software (BD Biosciences), respectively. The BCa cell counts were always normalized to a predetermined number of counting beads (424902, BioLegend, CA), and mean fluorescence intensity (MFI) ratios for each of the targets as mentioned above studied were assessed with respect to the corresponding isotype in the BCa-DiO^+^ cells. The data was analyzed using FlowJo program v10 (Ashland, OR).

### Lymphocyte Infiltration Characterization

Differences in lymphocyte infiltration into 3D-O scaffolds as a result of different oxygen content were assessed. First, BCa cells were cultured in 3D-O scaffolds for 4 days. On day 4, incubation media was removed from top of the matrices. Following media removal, PBMCs suspended in media were added on top of the matrix. Scaffolds were cultured for 3 more days, and analyzed at day 7. On day 7, 3D-O matrices were enzymatically digested with collagenase and PBMCs were isolated and surface-stained with the following antibodies: FITC conjugated anti-CD3 (300406, BioLegend, CA), PE-Cy5 conjugated anti-CD4 (300508, BioLegend, CA), APC-Cy7 conjugated anti-CD8 (344714, BioLegend, CA), APC conjugated anti-CD19 (302212, BioLegend, CA) and BV510 conjugated anti-CD45 (304036, BioLegend, CA). Infiltrated populations were characterized with manual gating, or combined datasets were down-sampled and subjected to dimensionality reduction using t-stochastic neighbor embedding (t-SNE) algorithm (Abdelmoula et al., 2016) or automatically defined with FlowSOM clustering algorithm (Potts et al., 2007). Mean absolute numbers of CD3+, CD4+, CD8+, and CD19+ cells (normalized to beads) were determined in each experimental group. To confirm differences in CD8+ infiltration as a result of oxygen content variations, the number of infiltrated CD8+ cells were imaged using confocal microscopy or IHC.

### Effect of Reversing Oxygen Deprivation Within 3D-O Matrices

To attribute oxygen deprivation as the primary intermediary for all observed differences, we used a HIF inhibitor as a single agent (PX-478, 5 μM). PX-478 inhibitor was tested in HIF-1α expression, BCa cell proliferation, collagen-I expression, and CD8+ infiltration. We further examined a selective, high-affinity human IgG1 mAb that blocks programmed cell death ligand-1 (PD-L1) binding to PD-1 (Durvalumab, 5 μM) to evaluate its role on CD8 infiltration by flow cytometry and IHC as previously described.

### Statistical Analysis

Experiments were performed in triplicates and repeated at least three times. Results are presented as mean ± standard deviation, and statistical significance was analyzed using Student’s *t*-test or one-way ANOVA; a *p*-value less than 0.05 was considered significant. Outliers were removed using the Interquartile Range (IQR).

## Results

### 3D-O Matrices Demonstrate the Development of Physio- and Pathophysiological Breast Oxygen Content

Plasma was engineered to create a gelatinous-like (Figure 1D) and porous (Figure 1E) 3D-O matrix. The pore size observed in these scaffolds was about 10 μm. pO_2_ levels were profiled from the top to bottom inside cell-seeded 3D-O models. After 4 days in culture, cell-seeded 3D-O matrices incubated at 21% O_2_ were found to exhibit a top pO_2_ value of 7.3 ± 1.3 kPa and a bottom value of 5.3 ± 2.6 kPa (Figure 1F). A gradual decrease in pO_2_ levels was manipulated by oxygen incubation in a hypoxic globe chamber. When the scaffolds were incubated at 1.5% O_2_ the pO_2_ levels developed within the scaffolds dropped to 0.7 kPa at the top to 0.4 kPa in the bottom (Figure 1F). As the pO_2_ differences between top and bottom of the 3D-O matrices were observed to be not significantly different, the average value for each condition was considered as reference. Hereafter, the 3D-O matrices will be referred to as 3D-O physiological (reflecting an average pO_2_ content of 6.3 ± 2.1 kPa) and 3D-O tumorous (reflecting an average pO_2_ content of 0.64 ± 0.08 kPa), respectively.

**FIGURE 2:**
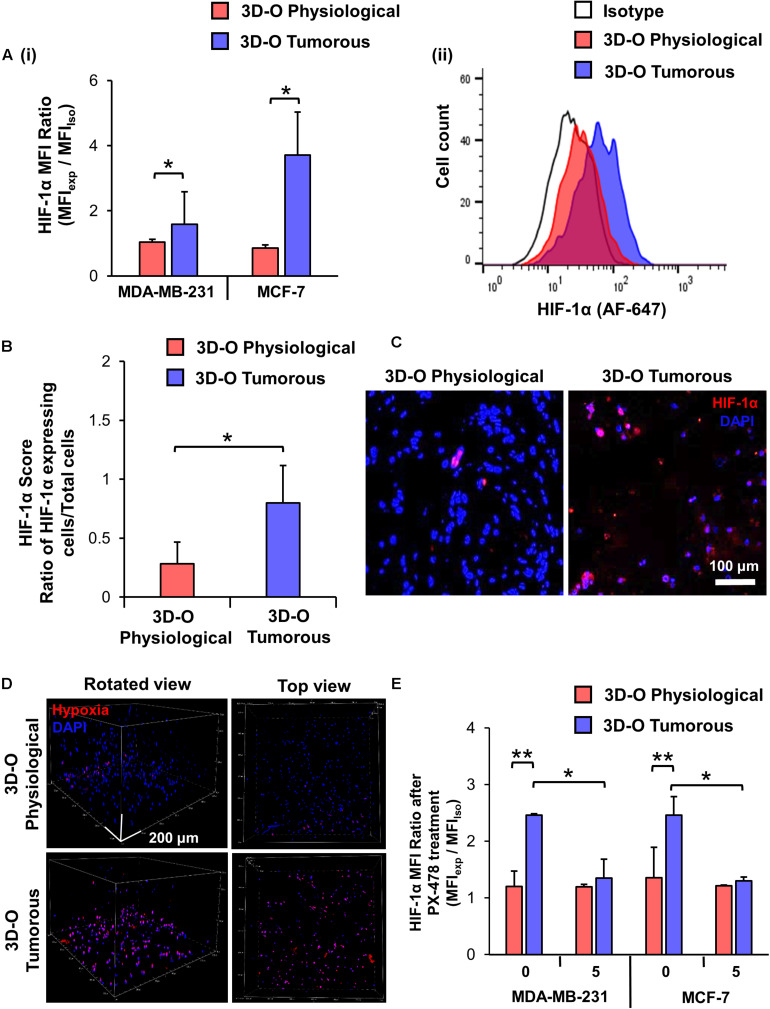
Validation of hypoxic phenotype in 3D-O matrices. **(A)** HIF-1α expression by MDA-MB-231 and MCF-7 cells grown in 3D-O physiological and 3D-O tumorous matrices after 4 days quantified as MFI ratio between AF647-anti-HIF-1α and AF647 isotype control **(i)** and flow cytometry representative histogram **(ii)**. **(B)** Mean HIF-1α score indicating the percentage of MDA-MB-231 cells positive for HIF-1α expression after 4 days in 3D-O physiological and 3D-O tumorous matrices. **(C)** Representative fluorescent imaging at day 4 for MDA-MB-231 cells grown within 3D-O physiological and 3D-O tumorous; DAPI: Blue; HIF-1α: Red. Merged: Pink. Scale bar = 100 μm. **(D)** A hypoxia-sensing dye was added to both 3D-O physiological and 3D-O tumorous matrices containing MDA-MB-231 cells on day 4. After 2 h, scaffolds were imaged using confocal microscopy to monitor hypoxia fluorescence marker associated with the BCa cells. Representative fluorescent images of z-stack images at day 4 for MDA-MB-231 cells grown within 3D-O physiological and 3D-O tumorous; DAPI: Blue; Hypoxia reagent: Red). Scale bar = 200 μm. **(E)** HIF-1α expression for MDA-MB-231 and MCF-7 cells after treatment with PX-478 at 5 μM concentration quantified as MFI ratio between AF647-anti-HIF-1α and AF647 isotype control in 3D-O physiological and 3D-O tumorous matrices after 4 days. **p < 0.001, *p < 0.05, data were analyzed using unpaired two-tailed Student’s *t*-test.

The effect of the reduction in oxygen content in BCa cells within 3D-O scaffolds was confirmed using confocal microscopy and a hypoxia-sensing dye (Image-iT Hypoxia Reagent). 3D-O tumorous matrices showed a significant increase in the number of BCa cells expressing HIF-1α, in which the HIF-1α MFI ratio was 1.6 and 3.7 times higher compared to 3D-O physiological matrices for MDA-MD-231 and MCF-7, respectively (Figure 2Ai). Representative flow cytometry histogram of Alexafluor 647-HIF-1α signal in 3D-O physiological and 3D-O tumorous compared to isotype control MDA-MB-231 cells showed an increase in HIF-1α expression in 3D-O tumorous compared to 3D-O physiological matrices (Figure 2Aii). Immunofluorescence of 3D-O physiological and 3D-O tumorous scaffold sections using anti-HIF-1α antibody revealed that the HIF-1α score (ratio of positive HIF-1α expressing cells/total cells) was significantly higher for MDA-MB-231 cells grown under oxygen-deprived conditions in 3D-O tumorous scaffolds compared to the cells grown in 3D-O physiological scaffolds, as illustrated in Figures 2B,C. A hypoxia reagent that fluoresces when oxygen levels fall below 5% confirmed that a higher number of MDA-MB-231 cells growing within 3D-O tumorous matrices exhibited positive fluorescent signals compared to the 3D-O physiological matrices (Figure 2D). The green fluorescence from the Hypoxia reagent was imaged using the confocal microscope to demonstrate the co-localization of the hypoxia stain (re-colored in red) and the DAPI (blue) of the cells. Despite some background as determined by only red stain, a pink color (co-localized red and blue) was selected as a positive signal, as illustrated in Supplementary Figure 1A. Significantly higher number of MDA-MB-231 cells (stained Blue with DAPI) grown within 3D-O tumorous scaffold (ratio of 0.61) compared to 3D-O physiological (ratio of 0.10) were stained with the hypoxia reagent, as illustrated in Supplementary Figure 1Bi. The Pearson coefficient for co-localization of hypoxia reagent stain and DAPI for these cells was also significantly higher in 3D-O tumorous (Pearson coefficient = 0.83) than those grown in 3D-O physiological matrix (Pearson coefficient = 0.44) (Supplementary Figure 1Bii). We then evaluated the effect of HIF inhibitor PX-478 (5 μM) on HIF-1 α expression under 3D-O physiological or tumorous conditions. PX-478 significantly decreased the expression of HIF-1α in both MDA-MB-231 and MCF-7 cells incubated in 3D-O tumorous conditions as compared to the level of 3D-O physiological conditions (Figure 2E).

### Low-Oxygen Within 3D-O Matrices Hinders BCa Cell Proliferation

**FIGURE 3:**
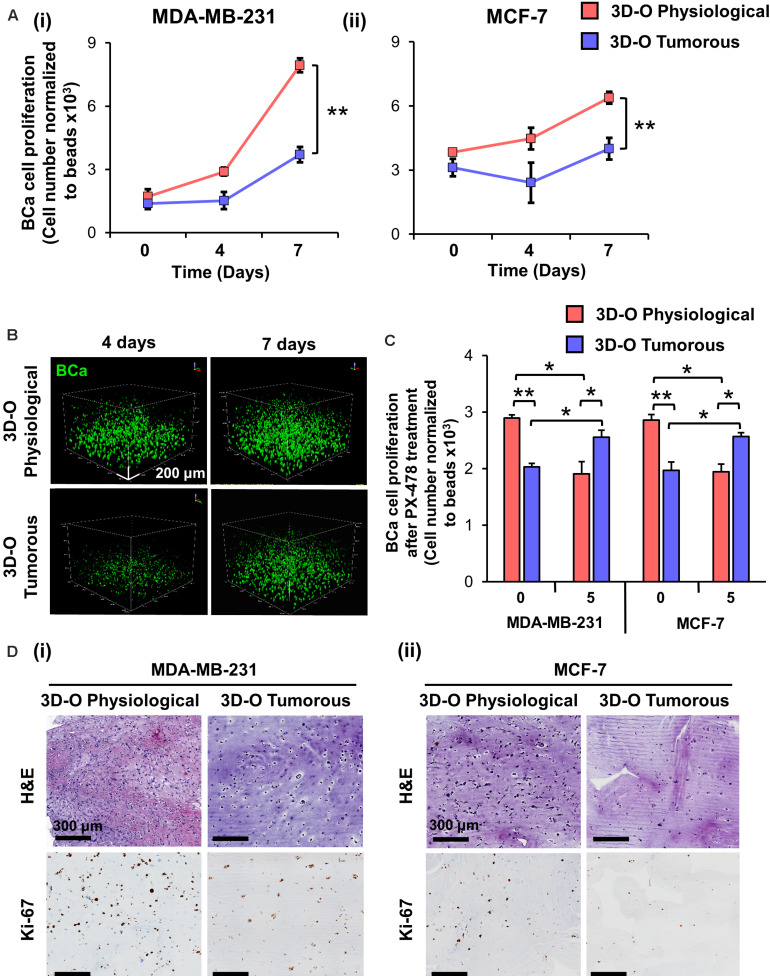
Low-oxygen within 3D-O matrices hinders BCa cell proliferation. **(A)** Effect of oxygen deprivation on the proliferation of MDA-MB-231 **(i)** and MCF-7 cells **(ii)** grown for 7 days either in 3D-O physiological or 3D-O tumorous matrices. **(B)** Representative confocal microscopy images of MDA-MB-231 cells (green) inside 3D-O matrices at days 4 and 7 represented by z-stack images from top to bottom in rotated view. Scale bar = 200 μm. **(C)** Proliferation of MDA-MB-231 and MCF-7 cells after treatment with PX-478 at 5 μM concentration quantified as cell numbers normalized to beads grown in 3D-O physiological and 3D-O tumorous matrices after 4 days. **(D)** H&E staining and IHC results representing Ki-67 expression in MDA-MB-231 **(i)** and MCF-7 cells **(ii)** grown for 4 days within 3D-O physiological or 3D-O tumorous matrices. Scale bar = 300 μm., **p < 0.001, *p < 0.05, data were analyzed using one-way analysis of variance (ANOVA), and unpaired two-tailed Student’s *t*-test.

To evaluate the impact of an oxygen-deprived environment on BCa proliferation, we analyzed BCa cell numbers in 3D-O physiological and tumorous matrices by flow cytometry. As illustrated in Figure 3A, for both MDA-MB-231 and MCF-7 cell lines, the rate of BCa cell proliferation was observed to be significantly hindered in 3D-O tumorous compared to 3D-O physiological model at days 4 and 7. These findings were confirmed by confocal imaging where BCa cell (DiO, green) proliferation was significantly diminished in 3D-O tumorous (262 ± 46 cells) when compared to 3D-O physiological (470 ± 61 cells) at day 4 and 3D-O tumorous (329 ± 39 cells) in contrast to 3D-O physiological (572 ± 46 cells) at day 7, respectively (Figure 3B and Supplementary Figures 2Ai,Aii). BCa cells were also observed to form significantly less clusters in 3D-O tumorous (49 ± 6 cell clusters) when compared to 3D-O physiological (74 ± 4 cell clusters) at day 4 and 3D-O tumorous (60 ± 1 cell clusters) in contrast to 3D-O physiological (77 ± 4 cell clusters) at day 7, respectively (Figure 3B and Supplementary Figures 2Bi,Bii). The effect of HIF inhibitor PX-478 (5 μM) on proliferation of both MDA-MB-231 and MCF-7 cells was evaluated and the results from both cell lines demonstrated that BCa cell numbers can be significantly restored under 3D-O tumorous scaffold conditions when HIF-1α is inhibited (Figure 3C). These findings confirmed that BCa cell proliferation could vary depending on the oxygen content in their surrounding matrix, as mimicked by our 3D-O platforms. We further corroborated the reduced capacity of MDA-MB-231 and MCF-7 cells to grow within 3D-O tumorous matrices compared to 3D-O physiological by H&E and Ki-67 staining, a marker of tumor cell proliferation (Figures 3Di,Dii). The number of eosin stained MDA-MB-231 and MCF-7 cells as well as those that exhibited positive Ki67 stains were also quantified from the IHC images by carefully assessing the difference between top and bottom of the 3D-O matrices. Similar results were found in the top vs. bottom areas of the matrix in terms of growth and proliferation for both cell lines. However, the results from these experiments demonstrated that cell proliferation and growth was significantly hindered under 3D-O tumorous compared to 3D-O physiological conditions measured by number of eosin stained cells and Ki67 positive staining (Supplementary Figures 3, 4).

### Low-Oxygen Within 3D-O Matrices Alters Matrix Environment and Cellular Marker Expression

To characterize the role of oxygen deprivation in the surrounding matrix, we studied the differences in expression of main fibrous

**FIGURE 4:**
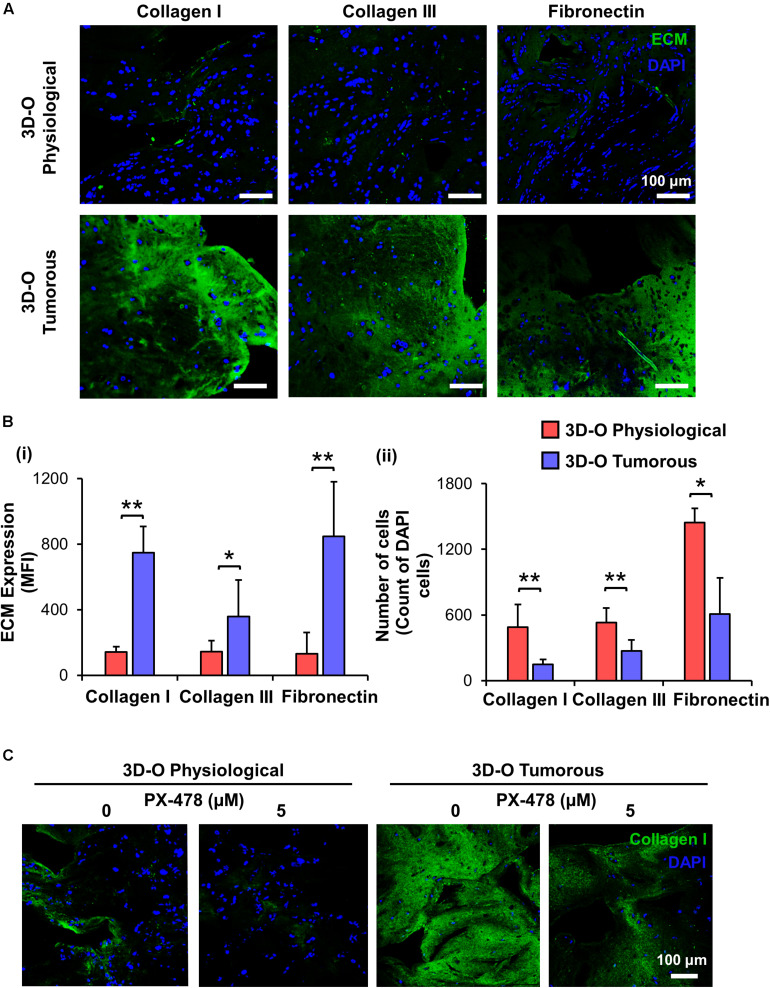
Low-oxygen within 3D-O matrices increases the expression of main fibrous ECM proteins. **(A)** Representative fluorescent images exhibiting changes in expression of collagen I, collagen III, and fibronectin at day 4 for MDA-MB-231 cells grown within 3D-O physiological and 3D-O tumorous matrices; DAPI: Blue; ECMs: green). Scale bar = 100 μm. **(B)** ECM expression by BCa cells growing within 3D-O physiological and 3D-O tumorous matrices, quantified as MFI of ECM expression **(i)** and the number of cells **(ii)**. **(C)** Representative fluorescent images exhibiting changes in collagen I expression by MDA-MB-231 cells grown in 3D-O physiological and 3D-O tumorous matrices after treatment with PX-478 for 4 days at 5 μM concentration. Scale bar = 100 μm. **p < 0.001, *p < 0.05, data were analyzed using unpaired two-tailed Student’s *t*-test.

ECM proteins associated with breast tissue including collagen I, collagen III and fibronectin by MDA-MB-231 cells under 3D-O tumorous and physiological conditions. Representative IF images showed a significant surge in expression of all the three studied ECMs in 3D-O tumorous as compared to the 3D-O physiological matrices (Figure 4A). Quantification of these fibrous ECM proteins indicated a significantly increased expression (Figure 4Bi), despite a much lower BCa cell count (Figure 4Bii) in 3D-O tumorous compared to 3D-O physiological models. As previously observed, oxygen deprivation-driven effects were reversed by blocking HIF-1α (PX-478, 5 μM). MDA-MB-231 cells grown within 3D-tumorous exhibited reduced collagen I expression when HIF was inhibited (Figure 4C).

**FIGURE 5:**
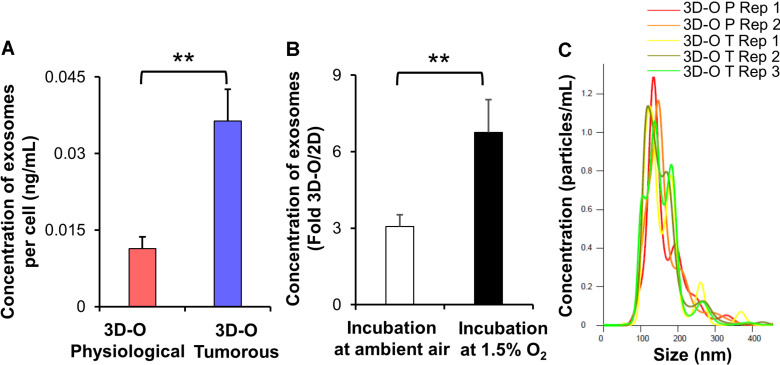
Low-oxygen within 3D-O matrices affects extracellular vesicle secretion. **(A)** The concentration of extracellular vesicles generated per MDA-MB-231 cell (ng/mL) grown within 3D-O physiological and 3D-O tumorous matrices for 4 days. **(B)** Fold change concentration of extracellular vesicles generated per cell (ng/mL) grown for 4 days either in 3D-O culture or matching traditional 2D culture and incubated either in ambient air or 1.5% O_2_. **(C)** Representative plot for extracellular vesicle size (nm) versus concentration (particles/ml) from 3D-O physiological and 3D-O tumorous matrices. **p < 0.001, data were analyzed using unpaired two-tailed Student’s *t*-test.

Additionally, we evaluated extracellular vesicle concentration under 3D-O tumorous and physiological conditions. We found that extracellular vesicle concentration under 3D-O tumorous was significantly increased compared to the 3D-O physiological model (Figure 5A). Additionally, extracellular vesicle content in 3D-O models was significantly (3 and 6.7-fold) higher than traditional 2D cultures under the same incubation regimens (ambient or 1.5% O_2_), respectively (Figure 5B). We further confirmed that extracellular vesicles did not show a change in size (± 100 nm) due to oxygen deprivation or 3D culture conditions (Figure 5C).

**FIGURE 6:**
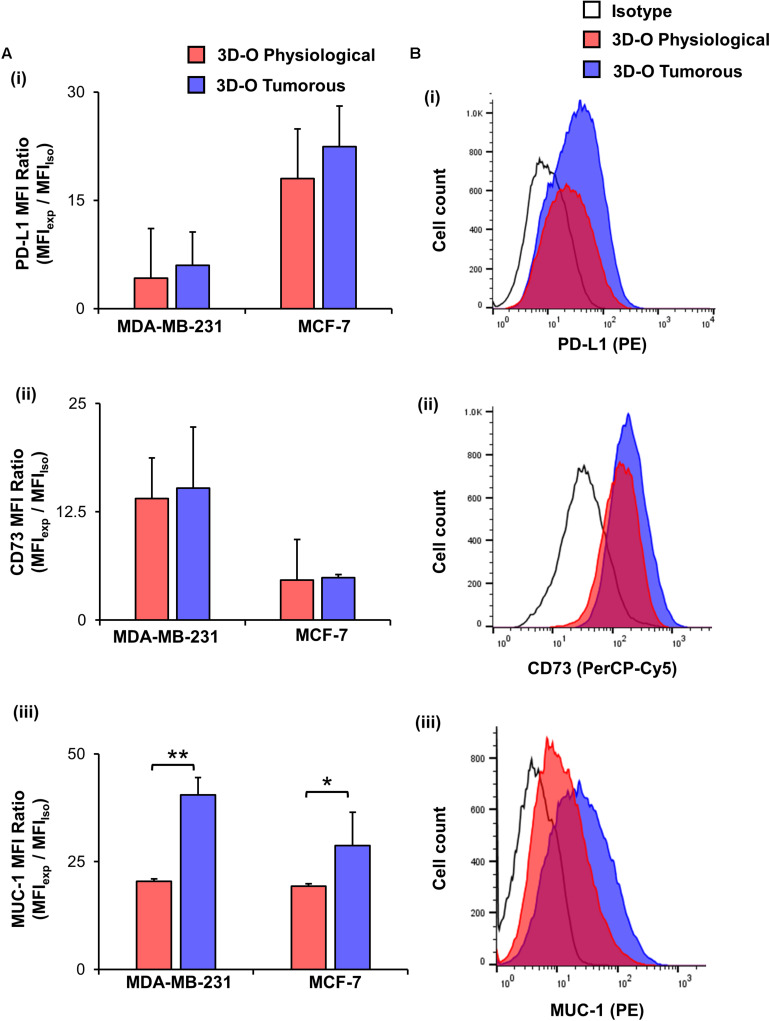
Low-oxygen within 3D-O matrices moderately enhances immune surface marker expression. **(A)** Immune surface marker expression by BCa cells grown in 3D-O physiological and 3D-O tumorous matrices after 4 days quantified as MFI ratio for PD-L1 **(i)**, CD73 **(ii)**, and MUC-1 **(iii)**. **(B)** Flow cytometry representative histograms for PD-L1 **(i)**, CD73 **(ii)**, and MUC-1 **(iii)** **p < 0.001, *p < 0.05, data were analyzed using unpaired two-tailed Student’s *t*-test.

Finally, to test the effect of oxygen deprivation in BCa immune-suppressive marker expression, we examined **PD-L1** (Figures 6Ai,Bi), CD73 (Figures 6Aii,Bii) and MUC-1 expression (Figures 6Aiii,Biii) by both MDA-MB-231 and MCF-7 cells cultured under 3D-O physiological and tumorous conditions. We found a modest increase in expression of the three immune-suppressive markers by BCa cells grown within 3D-O tumorous matrices as compared to 3D-O physiological models on day 4 for both MDA-MB-231 and MCF-7 cell lines.

### Low-Oxygen Within 3D-O Matrices Severely Reduces Lymphocyte Infiltration

**FIGURE 7:**
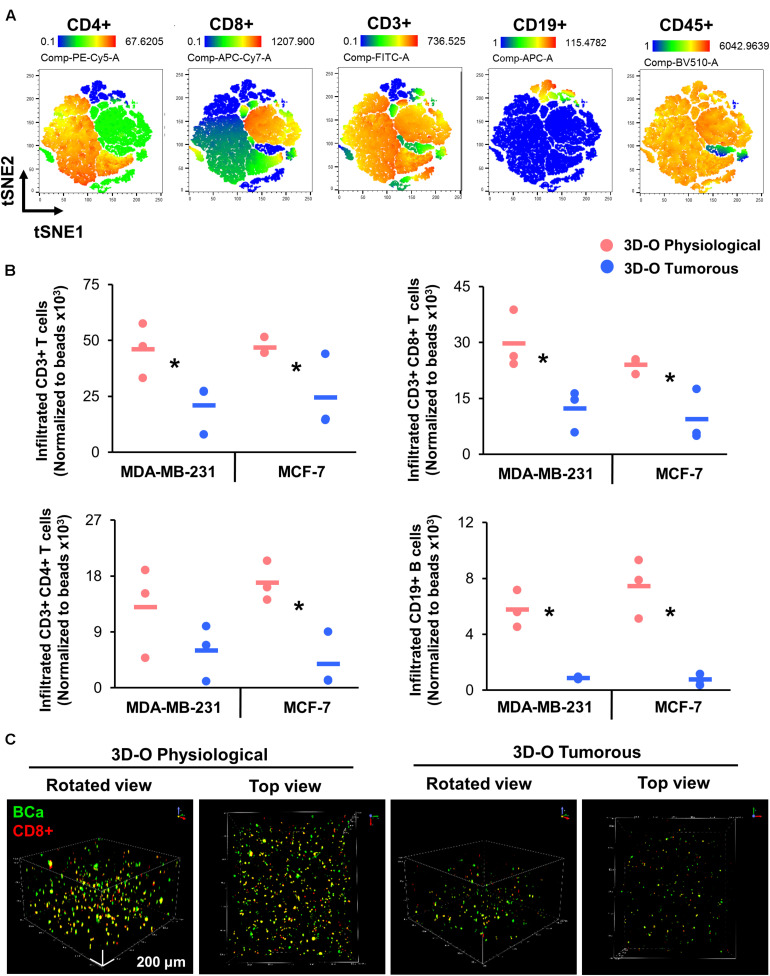
Low-oxygen within 3D-O matrices reduces lymphocyte infiltration. **(A)** tSNE plot of pre-processed, single, live cells, down-sampled to 10.000 events and concatenated showing major immune clusters (CD3-FITC+, CD4-PE-Cy5+, CD8-APC-Cy7+, CD19-APC+ and CD45-BV510+). **(B)** Quantification of the number of infiltrated lymphocytes into 3D-O physiological and 3D-O tumorous matrices embedded with either MDA-MB-231 cells or MCF-7 cells by manual gating. Infiltration data shown represents PBMCs obtained from 3 individual healthy subjects and the average of infiltrated CD3+ T cells, CD3+CD8+ T cells, CD3+CD4+ T cells, and CD19+ B cells (data normalized to counting beads x10^3^), *p < 0.05, data were analyzed using unpaired two-tailed Student’s *t*-test. **(C)** Representative confocal microscopy images of rotated and top views of BCa (green) cells and CD8+ (red) cells in 3D-O physiological and 3D-O tumorous matrices on day 7, co-localization is seen in yellow. Scale bar = 200 μm.

We further evaluated the effect of oxygen deprivation in lymphocyte infiltration into 3D-O physiological and tumorous scaffolds. We performed three independent analyses (manual gating, t-SNE and FlowSOM) to assess the effect of oxygen deprivation on lymphocyte infiltration into 3D-O models by flow cytometry. Manual gating allowed us to clearly identify infiltrated lymphocytes as CD3+ T cells (CD3-FITC+), CD4+ T cells (CD3-FITC+ CD4-PE-Cy5+), CD8+ T cytotoxic cells (CD3-FITC+ CD8-APC-Cy7+) and B cells (CD19-APC+) (Supplementary Figure 5A). The t-SNE algorithm was used to reduce the dimensionality of our flow cytometry results related to the phenotypes of infiltrating immune cells. A t-SNE algorithm was performed with different perplexity values to confirm the results. After data processing, t-SNE and FlowSOM algorithms were used to identify the major immune cell populations in a data-driven and automated manner. t-SNE (Figure 7A) and FlowSOM (Supplementary Figure 5B) assigned cells to clusters corresponding to the major immune clusters (CD3-FITC+, CD4-PE-Cy5+, CD8-APC-Cy7+, and CD19-APC+). FlowSOM cluster correlated with the same key populations (3 (BV510-high, FITC-high, PE-Cy5-high, APC-Cy7-low, APC-low), 2 (BV510-high, FITC-high, PE-Cy5-low, APC-Cy7-high, APC-low), 1 (BV510-high, FITC-high, PE-Cy5-low, APC-Cy7-low, APC-low) and 0 (BV510-high, FITC-low, PE-Cy5-low, APC-Cy7-low, APC-high). Visualization of the high-dimensional data corresponded well to the FlowSOM algorithm (Supplementary Figure 5C) and manual gating (Supplementary Figure 5D). Using this approach, we identified significantly impaired infiltration of CD3+, CD8+, CD4+, and CD19+ cells inside MDA-MB-231 and MCF-7 cell embedded 3D-O tumorous scaffolds as compared to 3D-O physiological scaffolds (Figure 7B). To confirm these findings, sections of MDA-MB-231 and MCF-7 cell containing 3D-O physiological and 3D-O tumorous matrices were probed with anti-CD3 and anti-CD44 (a known hallmark of BCa cells) via IHC staining (Supplementary Figures 6A, 7A). Quantification of the CD3 (Supplementary Figures 6Bi, 7Bi) and CD44 stain (Supplementary Figures 6Bii, 7Bii) in IHC full depth histological images confirmed the reduced cell number for CD3 cells in 3D-O tumorous compared to 3D-O physiological scaffolds. Similar results were found for top vs. bottom areas in terms of BCa CD44 cells presence and CD3 infiltration for both cell lines. We further confirmed oxygen-deprivation driven impaired CD8+ infiltration by confocal imaging of 3D-O physiological and 3D-O tumorous scaffolds that were embedded with MDA-MB-231 cells (Figure 7C). While CD8+ infiltration in 3D-O physiological matrices showed robust infiltration and clear co-localization of CD8+ (red) and BCa cells (green) (Supplementary Video 1), CD8+ infiltration in 3D-O tumorous matrices was hindered (Supplementary Video 2).

**FIGURE 8:**
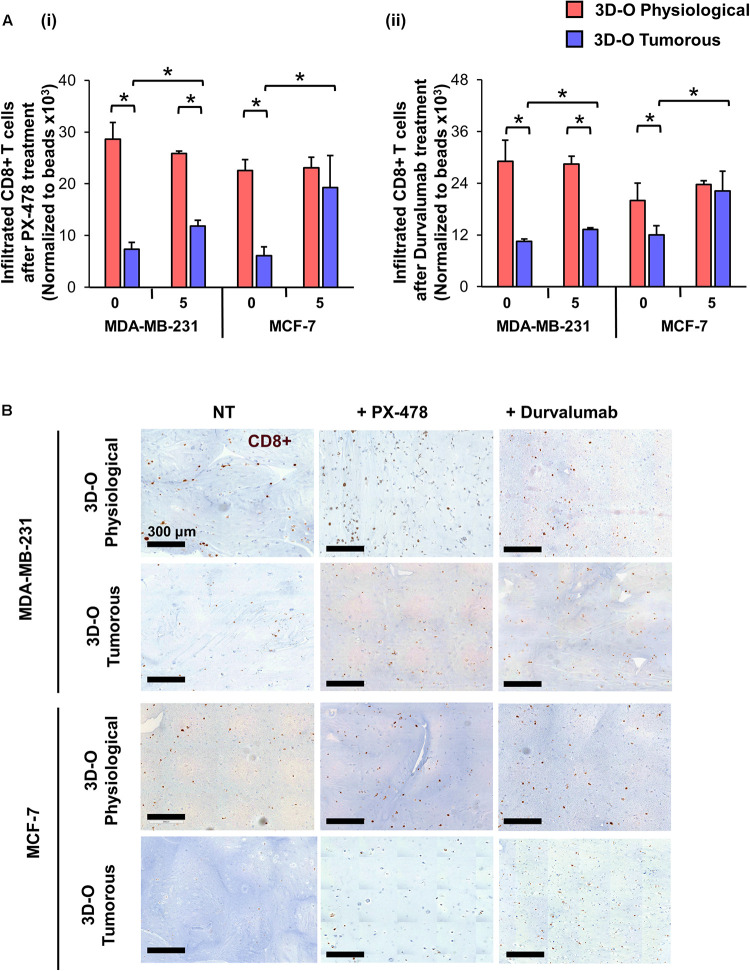
Sensitization of BCa cells to cytotoxic CD8+ T cells within 3D-O matrices. **(A)** CD8+ infiltration into 3D-O physiological and 3D-O tumorous matrices seeded with MDA-MB-231 and MCF-7 cells on day 7 after treatment with PX-478 at 5 μM concentration **(i)** and Durvalumab at 5 μM concentration **(ii)** for the first 4 days. **(B)** Representative IHC results representing CD8+ infiltration into 3D-physiological and 3D-tumorous scaffolds seeded with MDA-MB-231 and MCF-7 cells on day 7 after treatment with PX-478 and Durvalumab, Scale bar = 300 μm. *p < 0.05, (n.s.) not significant, data were analyzed using unpaired two-tailed Student’s *t*-test.

Moreover, the effect of the HIF inhibitor PX-478 (5 μM) on CD8 infiltration was evaluated using flow cytometry and IHC staining of matrices embedded with both MDA-MB-231 and MCF-7 cells (Figures 8Ai,B). The results demonstrated that CD8+ infiltration can be restored in 3D-O tumorous matrices as compared to the cell infiltration numbers into 3D-O physiological ones when HIF-1α is inhibited via PX-478 treatment (Figures 8Ai,B). Quantification of CD8 positive stain in IHC full depth histological images confirmed impaired infiltration of CD8 cells in 3D-O tumorous compared to 3D-O physiological scaffolds and treatment with PX-478 overcome those immune evasion mechanisms (Supplementary Figures 8A,B, 9A,B). Similar results were found in the top vs. bottom areas of the matrix in terms of CD8 infiltration for both cell lines (Supplementary Figure 8, 9). Additionally, we also found that treatment with an investigational anti-PD-L1 monoclonal antibody (Durvalumab, 5 μM) can also reverse CD8 infiltration in 3D-O tumorous matrices as compared to the cell infiltration numbers of the 3D-O physiological matrices (Figures 8Aii,B). These findings were corroborated by results from IHC staining (Supplementary Figures 8A,B, 9A,B). These findings correlate with our previous result showing that the expression of PD-L1 was enhanced in 3D-O tumorous matrices compared to 3D-O physiological matrices (Figures 6Ai,Bi).

## Discussion

A key hallmark of cancer is the tumor’s capacity to adapt to changeable states of oxygen deprivation. As oxygen content regulation is essential to maintaining cell homeostasis, small deviations in oxygen level within TME can result in significant changes in tumor cell functionality (Fathollahipour et al., 2018). Oxygen fluctuation occurs at irregular intervals in cancer with sporadic reoxygenation periods, but undoubtedly, oxygen depletion in tumors directly affects clinical responses to therapy by influencing tumor growth and is associated with a more aggressive tumor phenotype (Rofstad et al., 2010; Bayer and Vaupel, 2012). Mounting evidence from cancer research demonstrates that oxygen deprivation significantly promotes stark differences in cancer behaviors including tumor progression, drug resistance and immunosuppression (Harris, 2002; Eales et al., 2016). There is a growing need to develop new strategies to better reproduce *in vitro* the physical and chemical properties of the *in vivo* TME and thus to investigate tumor and immune system biology under well-controlled and more realistic environmental conditions. To better understand the role of the TME on variations of cancer development it is necessary to perform experiments under conditions that account for physio- or pathophysiological *in vivo* oxygen levels. Therefore, the use of *in vitro* models that can recreate applicable oxygen availability might be critical.

The challenges involved with the translation of bench therapies to clinical outcomes perhaps originate from a limitation of suitable models to adequately mimic the native oxygen-deprived TME in its entirety (Langhans, 2018). Recreating a relevant oxygen profile in current cancer models might be essential for determining tumor-immune evasive mechanisms. Very few models have looked at deciphering the interactions between oxygen deprivation and immune evasion inside the breast tumor. There are some studies whose experimental settings address some of these key facets. Oxygen diffusibility and consumption within a 3D ECM niche was previously explored by Colom et al. using a mathematical model (Colom et al., 2014). Crosstalk of macrophages and cancer cells was shown to be influenced by oxygen availability (Campillo et al., 2019). In another study, immune cell infiltration into 3D matrices was shown to vary significantly based on the oxygen content, when cancer cells and highly efficient chimeric antigen receptor T (CAR-T) cells were incorporated *in vitro* in a 3D micropattern within a photo-crosslinked hydrogel and coupled to a microfluidic hypoxia device (Ando et al., 2019). In this regard, we developed a gelatinous-like and porous 3D *in vitro* model recapitulating key oxygen levels allowing us to explore further the role of oxygen availability in tumor-immune interactions. The 10 μm pore size for the model reported in this study was promising to support immune cell migration through its fibers.

Physiological oxygen levels in solid tumors are very heterogeneous ranging from 0.5 to 4% oxygen saturation compared to 4–14% in healthy tissues (Vaupel et al., 2007; Carreau et al., 2011; McKeown, 2014; Muz et al., 2015). In addition, circulating and immune cells are exposed to a dynamic range of O_2_ content, in which the pO_2_ values vary from 13.5% in alveoli and 9.5% in arterial blood to 6.5% at the venous end of circulation (Tsai et al., 2003; McKeown, 2014; Lakshmanan et al., 2016; Farré et al., 2018; Keeley and Mann, 2019). After 4 days of culture the 3D-O physiological platform, exhibited an average pO_2_ of 6.3 ± 2 kPa mimicking physiological oxygen levels of healthy tissue and blood physiological levels that circulating and immune cells are exposed to. While 3D-O tumorous demonstrated pO_2_ 0.64 ± 2 kPa reflecting the transitioning pathophysiological oxygen levels occurring in tumor tissue.

The 3D-O model is developed from naturally occurring patient-derived resources minimizing the introduction of foreign elements that can alter the natural pathophysiology of the TME. During the blood coagulation process, fibrinogen, which is abundantly found in blood plasma, is converted to fibrin by the action of thrombin in a reaction highly dependent on calcium (Clark, 2001). There are several advantages of developing a platform with fibrin-rich plasma as the framework. Being enriched with growth factors and proteins that contribute to the regenerative process, a plasma-derived microenvironment potentiates cell proliferation, migration, and differentiation mimicking *in vivo* conditions, hence enhancing the clinical utility of the model (Zhao et al., 2007; de la Puente et al., 2015). Of note, the use of human plasma as a matrix not only supports cell structure, but also serves as a reservoir for nutrients, growth factors, cytokines, extracellular vesicles, and signaling molecules (de la Puente et al., 2013; de la Puente and Ludena, 2014; Kuznetsov et al., 2018) that help with the development of more relevant human cancer models. Variations in plasma fibrin content in between patients or other species like mouse and rat could be alluded as a limitation of employing plasma-derived platforms. A lower or higher fibrin content could result in constant modification of the amount of cross linkers, stabilizers and associated reagents to compensate platform integrity and functionality. In addition, as the native breast cancer environment is collagen I rich (Kauppila et al., 1998; Badaoui et al., 2017), our model does not provide an initial collagen-enriched environment. However, our fibrin-based matrix provides a “blank slate” for quantifying ECM expression/deposition. Despite this shortcoming, utilizing plasma is very cost-effective and helps conserve accessibility and safety by using the patient’s intrinsic constituents. In the last few years, many 3D models have employed plasma-based biomaterials, with both structural and functional purposes (Matsumoto et al., 2007; de la Puente et al., 2015; Tang et al., 2017).

In our studies, 3D-O matrices were enzymatically digested with collagenase and viability of the cellular populations was assessed to corroborate that this process did not affect cellular health (Data not shown). The results from our studies showed that the 3D-O model while supporting BCa cell growth, simulated physiological and pathophysiological oxygen levels leading to the generation of intratumoral hypoxic hallmarks such as increased HIF expression. Upregulated HIF expression is a well-known parameter associated with hypoxia and is known to affect several downstream biochemical processes (Jun et al., 2017). Our results confirm that oxygen deprivation within 3D-O matrices can efficiently reiterate HIF-driven regulation in the resident BCa cells. It is well known that within the hypoxic tumor niche, a high level of HIF-driven adaptations is observed in terms of ECM modeling (Gilkes et al., 2014), expression of immune surface markers PD-L1 (Noman et al., 2014), MUC-1 (Chaika et al., 2012), CD73 (Lan et al., 2018) and hypoxia-induced extracellular vesicles (Ren et al., 2019). Under oxygen deprivation in 3D-O tumorous matrices while BCa cell proliferation was subsided, extracellular vesicle secretion and ECM expression by cultured BCa cells were increased corroborating previous published findings. 3D-O scaffolds demonstrated a higher amount of secreted extracellular vesicles when compared to 2D and these changes in 3D-Os were further enhanced after oxygen deprivation. Extracellular vesicle yield in 3D cultures has already been shown to be higher than in 2D models (Haraszti et al., 2018), as well as that hypoxic conditions can lead cells to express more functionally potent vesicles (Almeria et al., 2019). Collagen type I is abundantly available and has been reported to be one of the critical breast cancer prognostic markers for clinical outcome (van ’t Veer et al., 2002). Hence, we evaluated whether HIF-1α blockage (PX-478, 5 μM), could reduce collagen I expression by BCa cells. The results from these experiments confirmed that inhibition of HIF-1α resulted in decreased collagen I deposition. Furthermore, the increased ECM expressions in response to oxygen deprivation could either be attributed to an absolute increase in new ECM generation or by remodeling and re-alignment of the native ECMs in the matrix environment (Chen et al., 2019). The low pO_2_ content within 3D-O tumorous scaffolds could be attributed for driving the differences in immune cell infiltration via enhanced ECM generation, slow BCa proliferation or a combination of both. By readapting the tumor microenvironment in absence of adequate oxygen, 3D-O tumorous scaffolds could also reflect oxygen diffusion impedance. Our data suggests that the low oxygen content within 3D-O tumorous scaffolds in association with other factors such as increased ECM and a change in the immunosuppressive secreted factors in the media could be functioning as a barrier for immune cell trafficking. Future experiments that look to systematically quantify low oxygen driven ECM deposition and secreted factors within the 3D-O scaffolds are needed to substantiate these claims conclusively. Alternatively, there could be other changes initiated by the observed low pO_2_ content in 3D-O tumorous scaffolds that might be affecting BCa cell characteristics such as a difference in nutrients, pH, and accumulation of metabolites, carbon dioxide gradient, and extracellular conditions. Further experiments will also be needed to support these predictions.

Breast cancer tumors comprise a highly inflammatory microenvironment supported by infiltrating immune cells, cytokines, and growth factors (Polyak, 2011). Moreover, the importance of boosting TILs in disease management has been cited as a critical therapeutic strategy (Kurozumi et al., 2019). Among TILs, CD8+ T cells have been shown to migrate and infiltrate tumors and mediate anti-tumoral responses (Dudley et al., 2002). Significant crosstalk exists between hypoxia and antitumor immune functions, where tumor hypoxia contributes to attenuated antitumor responses (Chang et al., 2019). There is evidence that oxygen deprivation influences many immune cells, including T cells and B cells (Sitkovsky and Lukashev, 2005). HIF-1α expression in cancer cells has been linked to the reduced ability of CD8+ T cells to recognize malignancy and lower the ability of T cells to proliferate (Lukashev et al., 2006). The ability to mimic tumor-mediated immune escape mechanisms driven by oxygen availability could be functionally fundamental for selecting the best strategies to combine therapies that have the maximum therapeutic potential (Odunsi, 2017). We further demonstrated that BCa cells grown within the oxygen deficient-niche of 3D-O tumorous scaffolds could promote tumor-immune evasive events. CD8+ T cells infiltration was significantly impaired under pathophysiological oxygen levels in the 3D-O tumorous model. HIF-1α and PD-L1 inhibition, re-sensitized BCa cells to cytotoxic CD8+ T cell infiltration. Previous studies have addressed management of the hypoxic tumor using HIF and PD-L1 inhibitors, which resulted in promising outcomes (Welsh et al., 2004; Noman et al., 2014; Zhu et al., 2017). It is also well established that boosting CD8+ cell infiltration into the tumor niche can reverse immune evasion (Maimela et al., 2019). In this context, a very promising PD-L1 checkpoint inhibitor (Durvalumab) has been well investigated to treat BCa tumors where CD8 infiltration has been challenging (Steele et al., 2018; Chia et al., 2019). Durvalumab is a fully human high affinity immunoglobulin monoclonal antibody that blocks PD-L1 by binding to PD-1 as reflected by its reported IC_50_ values (PD-1: 0.1nmol/l) and (CD-80: 0.04nmol/l). Another advantage of Durvalumab is that it binds specifically to PD-L1 but not to PD-L2, thus ensuring toxicity is limited as PD-L2 is known to regulate normal tissue inflammation (Botticella et al., 2019). Results from our experiments using Durvalumab on 3D-O matrices demonstrated these claims. The flow cytometry and IHC data reported in this study exhibited that treatment with Durvalumab can reverse and rescue CD8 infiltration in 3D-O tumorous matrices. These results complement our surface marker flow cytometry data that demonstrated that expression of PD-L1 was enhanced in 3D-O tumorous matrices compared to 3D-O physiological matrices. The clinical value of assessing tumor hypoxia is becoming more compelling as researchers can now successfully identify high-risk “hypoxic” patient subgroups as candidates for alternate therapy management strategies, which may include hypoxia-modification therapy (Walsh et al., 2014; Yang et al., 2018). Recognizing the critical need for hypoxia-induced PD-L1 expression assessment for patient stratification will reveal how targeted therapy (such as durvalumab) could benefit patients with hypoxia-induced PD-L1-expressing tumors. Further studies will be needed in order to validate this and other novel targeted modalities including combination therapy of hypoxia and PD-L1 inhibition.PX-478 can suppress tumor growth both *in vitro* and *in vivo* (Zhu et al., 2017), and its apoptosis-promoting and growth suppressor activities have been investigated in several different experimental models, including BCa cells (Welsh et al., 2004). However, in our experiments we have used the drug PX-478 at a very low dose in contrast to the studies that have shown these effects. Hence, further experiments are needed to investigate the role of this drug at higher doses within our experimental setup to ascertain how it changes behavior of BCa cell grown within 3D-O matrices and also establish if the drug-specific therapeutic outcomes can be recapitulated within the 3D-O platform.

**FIGURE 9:**
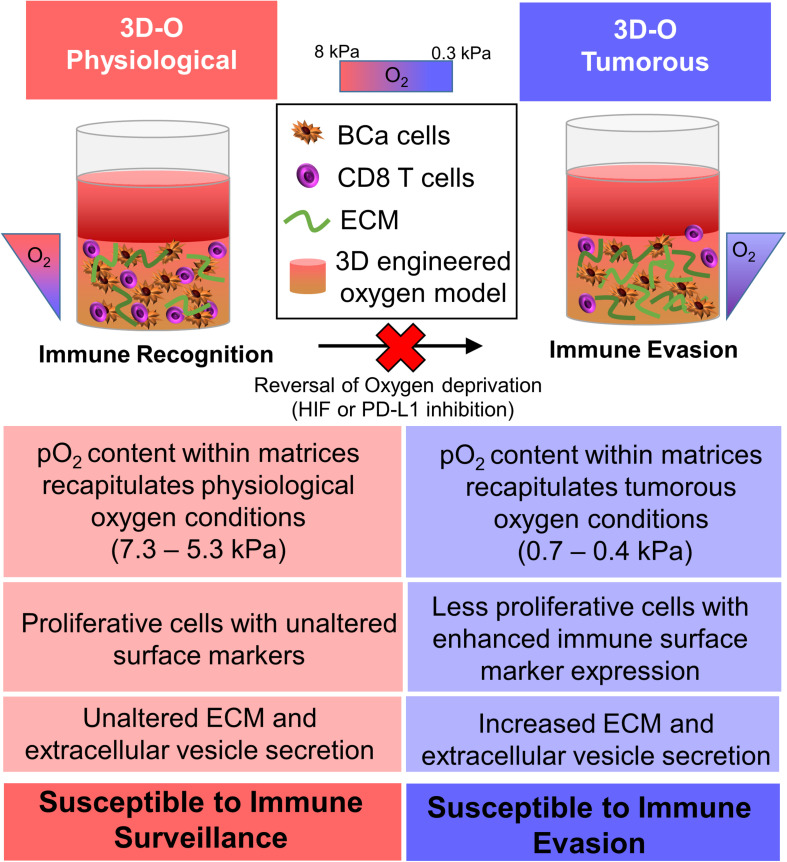
Graphical abstract. The 3D-O model generates physio- (3D-O physiological) and pathophysiological (3D-O tumorous) oxygen levels to understand the role of oxygen availability in tumor-immune interactions. Low oxygen-induced changes within the 3D-O tumorous model supported reduced BCa cell proliferation, increased extracellular matrix protein expression, increased extracellular vesicle secretion, enhanced immune surface marker expression on BCa cells, leading to immune evasion mechanisms compared to the 3D-O physiological model.

To summarize, we demonstrated that the engineered 3D platform, 3D-O, can efficiently mimic the microenvironment of the oxygen-deprived breast cancer tumor while accommodating pO_2_ content specific to the healthy and tumorous breast tissue. The 3D-O platform could serve as an efficient model that helps recreate tumor hypoxia driven immune evasion thus allowing investigation of the effects of an oxygen deficient BCa tumor on the cancer’s immune evasion machinery as close as possible to *in vivo* conditions. The 3D-O model generates physio- and pathophysiological oxygen levels to understand the role of oxygen availability in tumor-immune interactions. Insufficient pO_2_ content within the 3D-O tumorous model resulted in reduced BCa cell proliferation, increased extracellular matrix protein expression, increased extracellular vesicle secretion, enhanced immune surface marker expression on BCa cells, as well as immune evasion mechanisms compared to 3D-O physiological model (Figure 9). The significance of the 3D-O model is in its utility to perform as a highly maneuverable tool, which predicts accurate higher-throughput results for testing cancer drug efficacy. This utility is also significant because with this platform it would be possible to study previously not well-understood interactions between the low oxygen breast TME and the tumor’s immune evasion. Our approach advocates to significantly improving the understanding of how immune cells adapt to oxygen-deprived microenvironments and how that might affect tumor-immune interactions. The 3D-O model will allow us to study novel targeted therapies that aim to overcome hypoxia-driven immune evasion. The clinical value of assessing oxygen deprivation will reveal how hypoxia-modification therapy could benefit patients with hypoxic tumors (Walsh et al., 2014). As hypoxia within the tumor is the cause of several therapeutic discrepancies, a hypoxia specific approach would perhaps be practical to study tumor-immune interactions. Further studies will characterize the functionality of infiltrated immune cell populations within the oxygen-deprived environment. Additionally, patient-isolated cancer and accessory cells from a tumor biopsy will be incorporated into 3D-O matrices made of the same patient’s plasma to assess the depth-driven oxygen effects in the presence of immunomodulatory drugs and TME components in order to establish personalized studies. Experiments that look to evaluate how lymphocyte infiltration rate is modulated with respect to spatial differences within the 3D-O scaffolds could also reveal critical cues about the role of various immunosuppressive and immunomodulatory mechanisms at play within 3D-O cultures. In conclusion, the 3D-O model exhibited breast tissue-like physio- and pathophysiological oxygen levels that warranted further characterization of the low oxygen-induced changes in BCa cells including impaired cell proliferation, altered TME and hindered immune infiltration. Results from this study indicate that the 3D-O model could serve as a compelling platform for the evaluation of tumor-immunological events and as a drug-screening platform tool to overcome hypoxia-driven immune evasion.

## Data Availability Statement

The raw data supporting the conclusions of this article will be made available by the authors, without undue reservation.

## Ethics Statement

The studies involving human participants were reviewed and approved by Sanford Health Institutional Review Board. The patients/participants provided their written informed consent to participate in this study.

## Author Contributions

SB: methodology, investigation, validation, formal analysis, visualization, writing – original draft, and writing – review and editing. KC and MP: investigation, validation, and writing – review and editing. CE: resources and writing – review and editing. PP: conceptualization, methodology, visualization, writing – original draft, and writing – review and editing, supervision, project administration, and funding acquisition. All authors contributed to the article and approved the submitted version.

## Conflict of Interest

PP is co-founder of Cellatrix LLC, however, there has been no contribution of the aforementioned entity to the current study. PP, SB, and KC have a provisional patent application on the method described in this manuscript. The remaining authors declare that the research was conducted in the absence of any commercial or financial relationships that could be construed as a potential conflict of interest.
