# Supplementary figures and images for: Bioengineering the Oxygen-Deprived Tumor Microenvironment Within a Three-Dimensional Platform for Studying Tumor-Immune Interactions
Source: Front Bioeng Biotechnol. 2020 Sep 4;8:1040. doi: 10.3389/fbioe.2020.01040 (PMC7498579; doi:10.3389/fbioe.2020.01040)

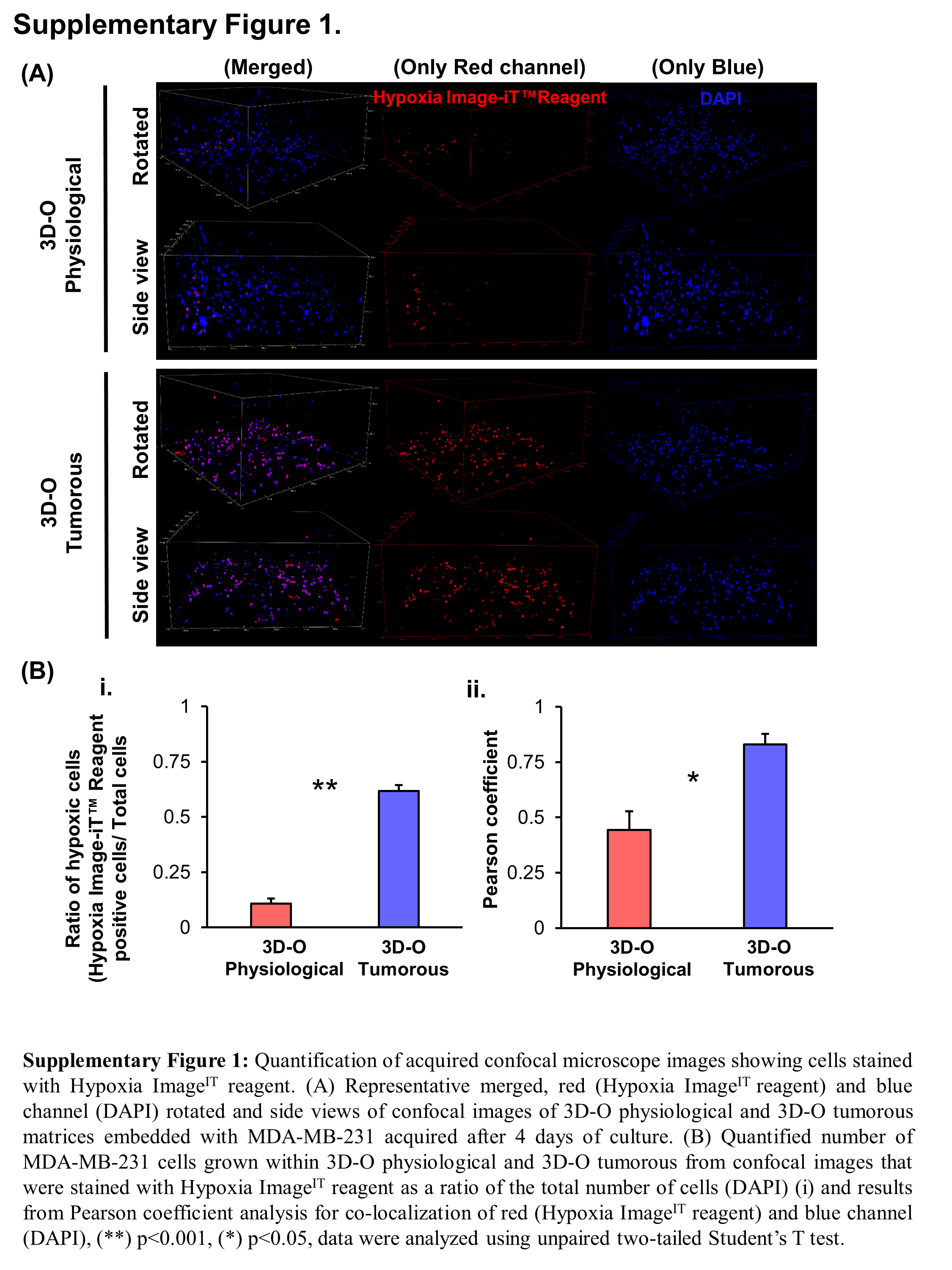

Supplement: Supplementary file 1 [file Image_1.tif]

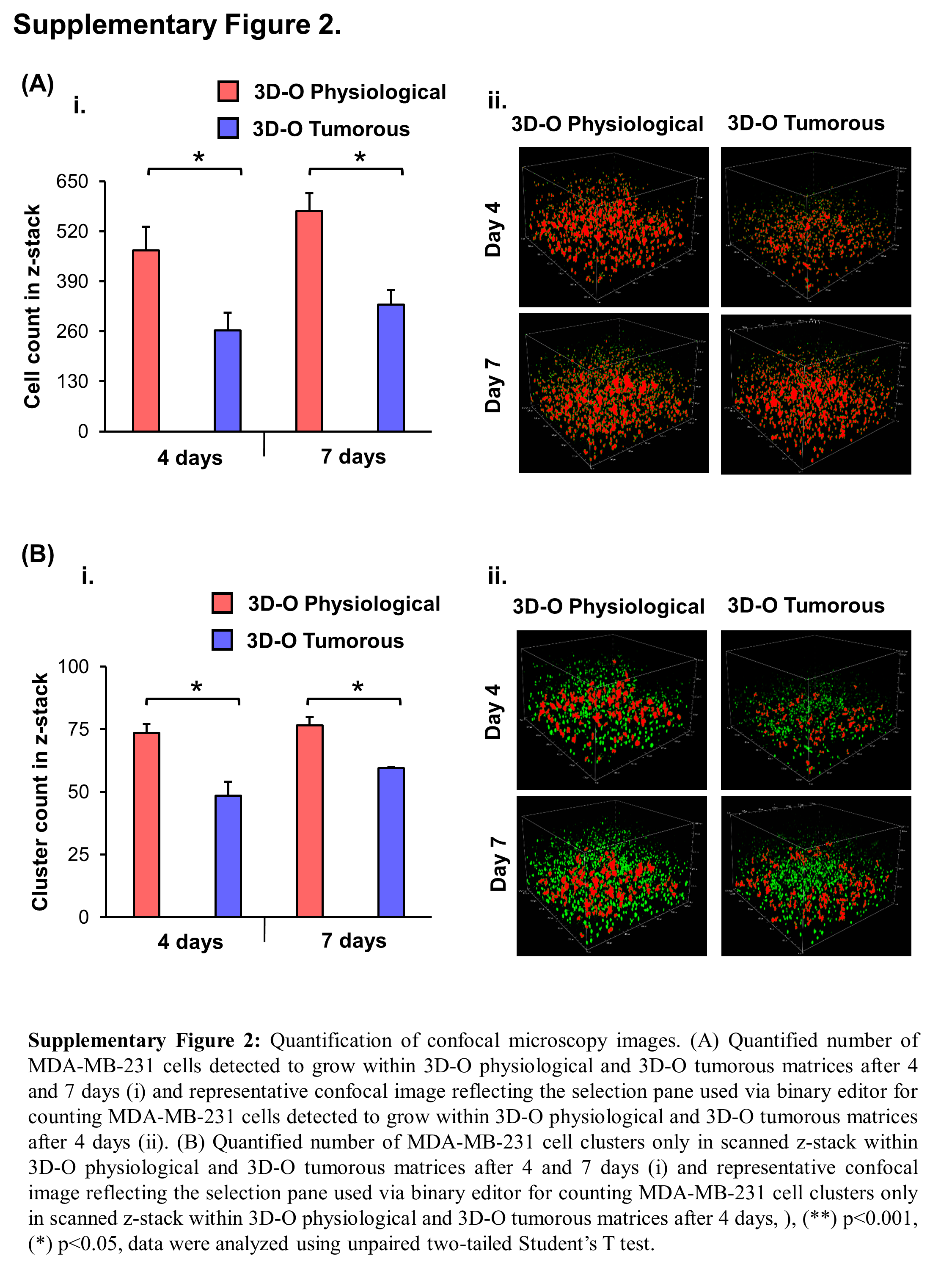

Supplement: Supplementary file 2 [file Image_2.TIF]

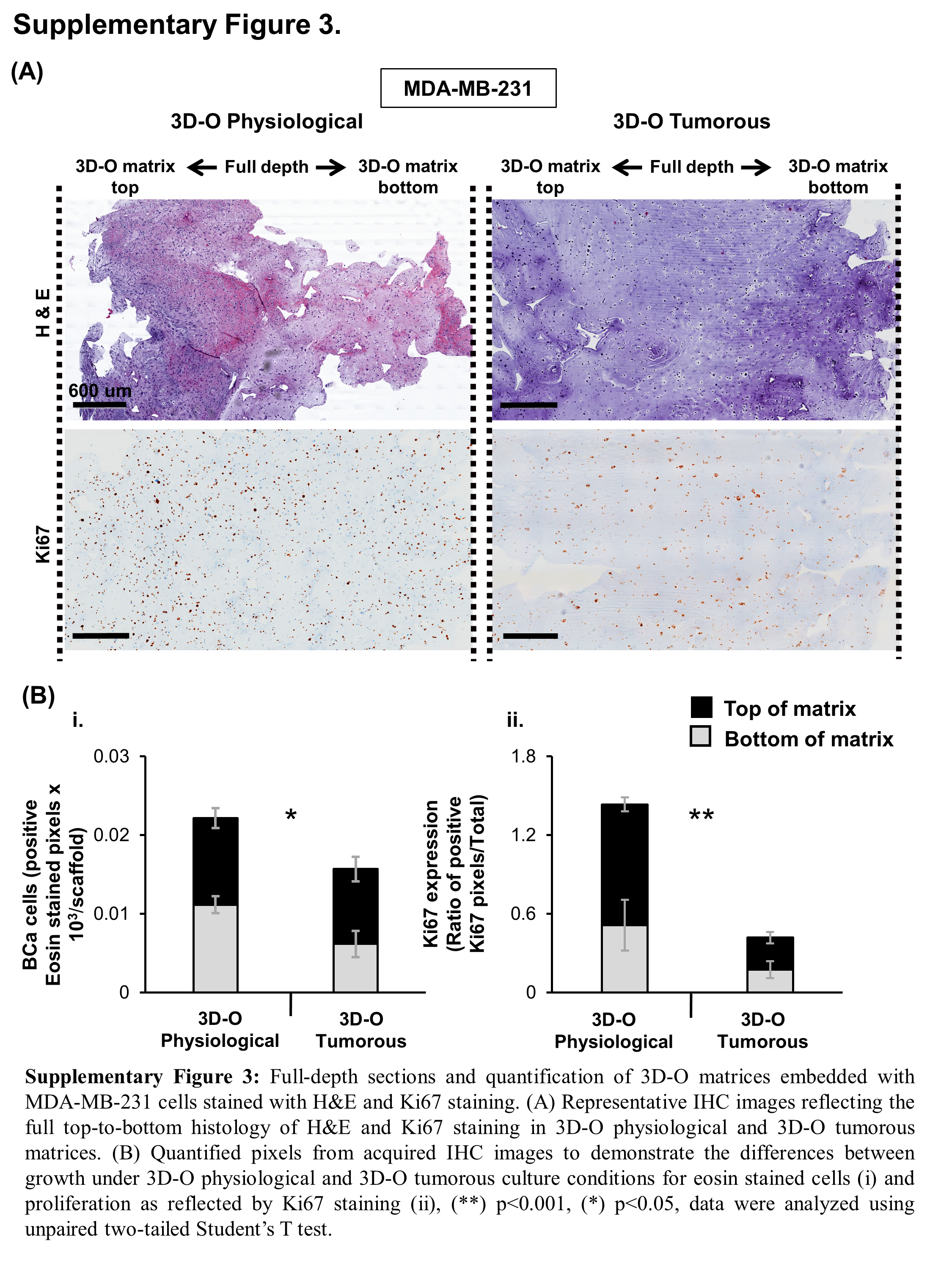

Supplement: Supplementary file 3 [file Image_3.TIF]

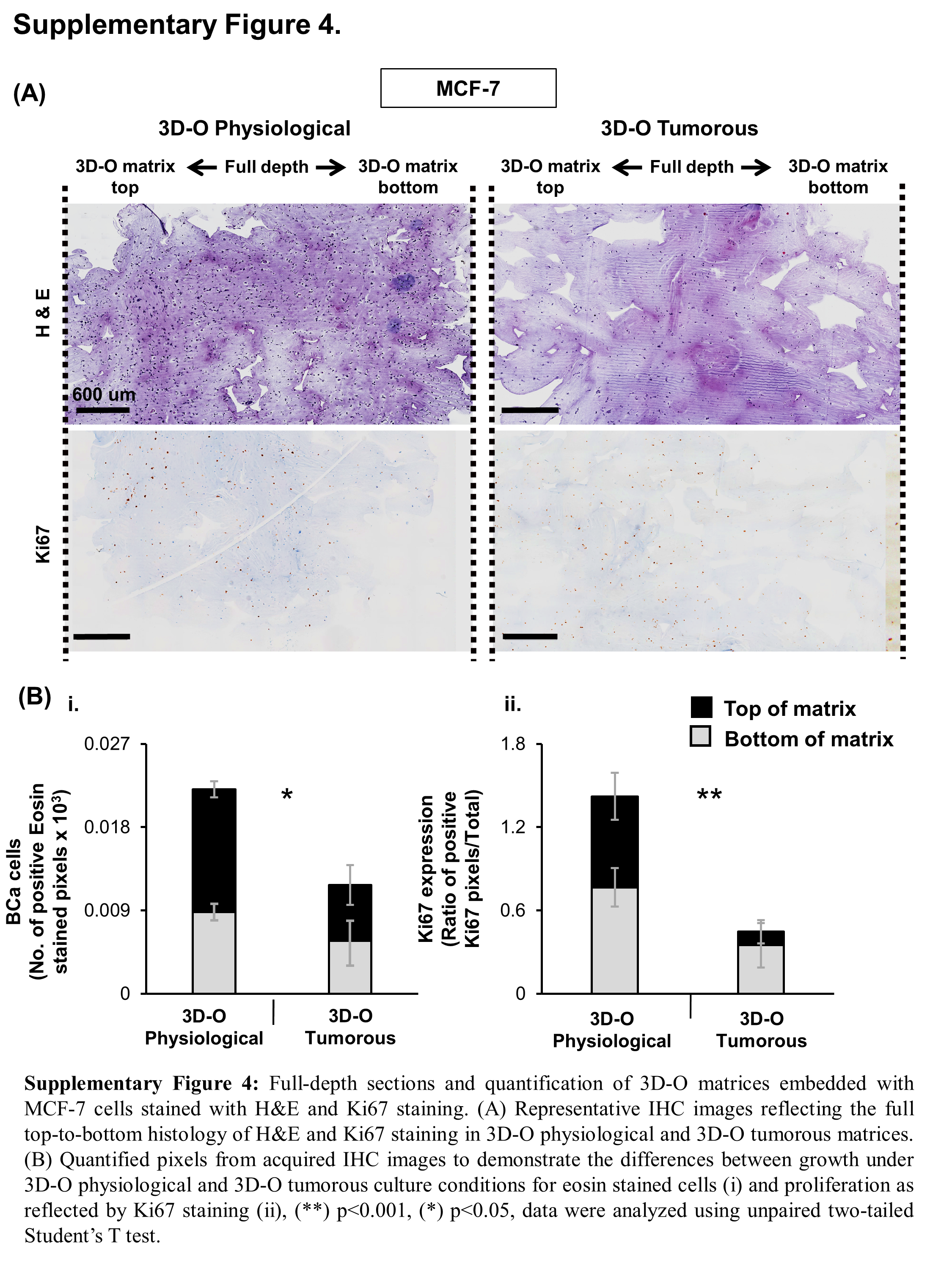

Supplement: Supplementary file 4 [file Image_4.TIF]

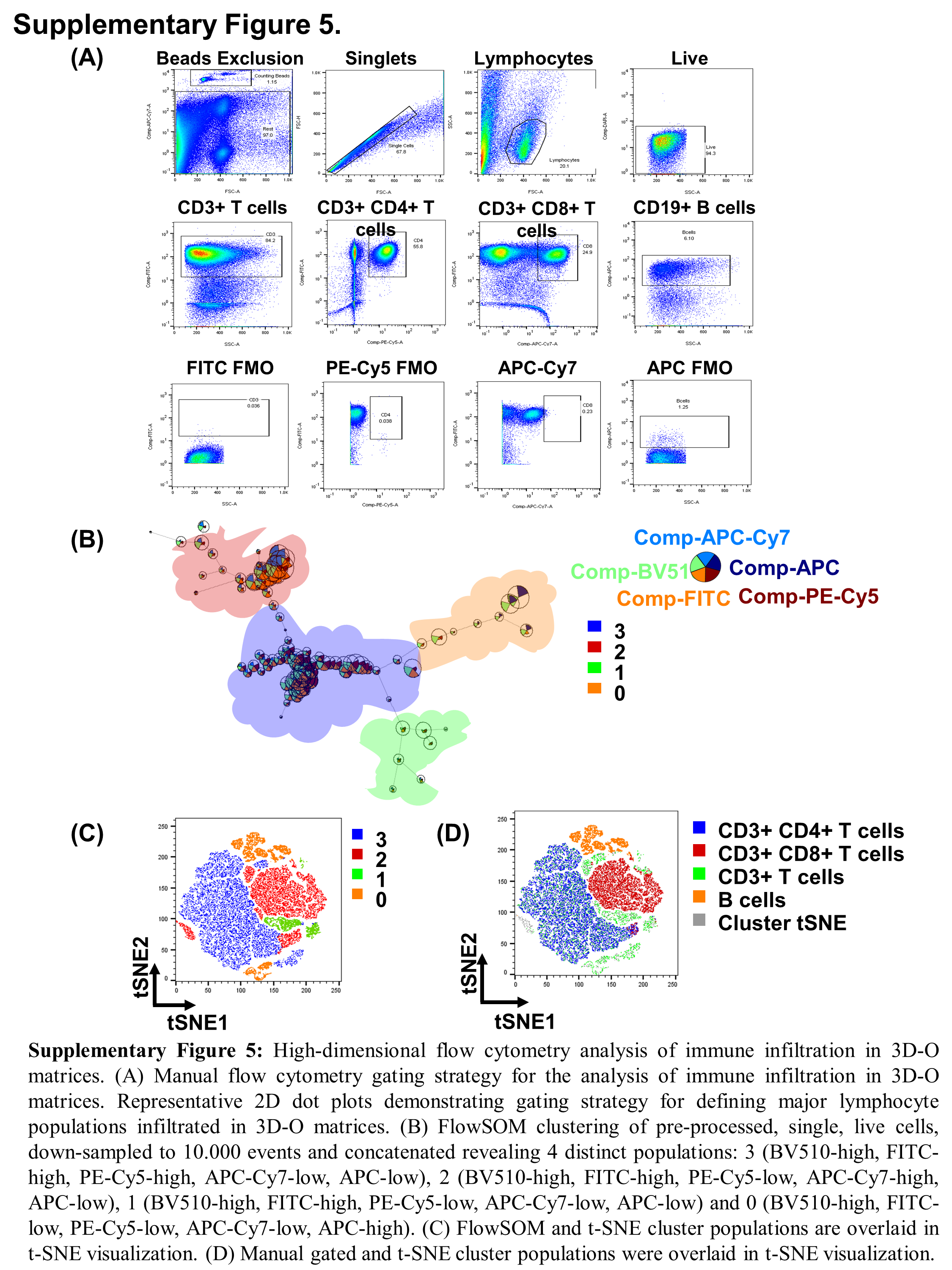

Supplement: Supplementary file 5 [file Image_5.TIF]

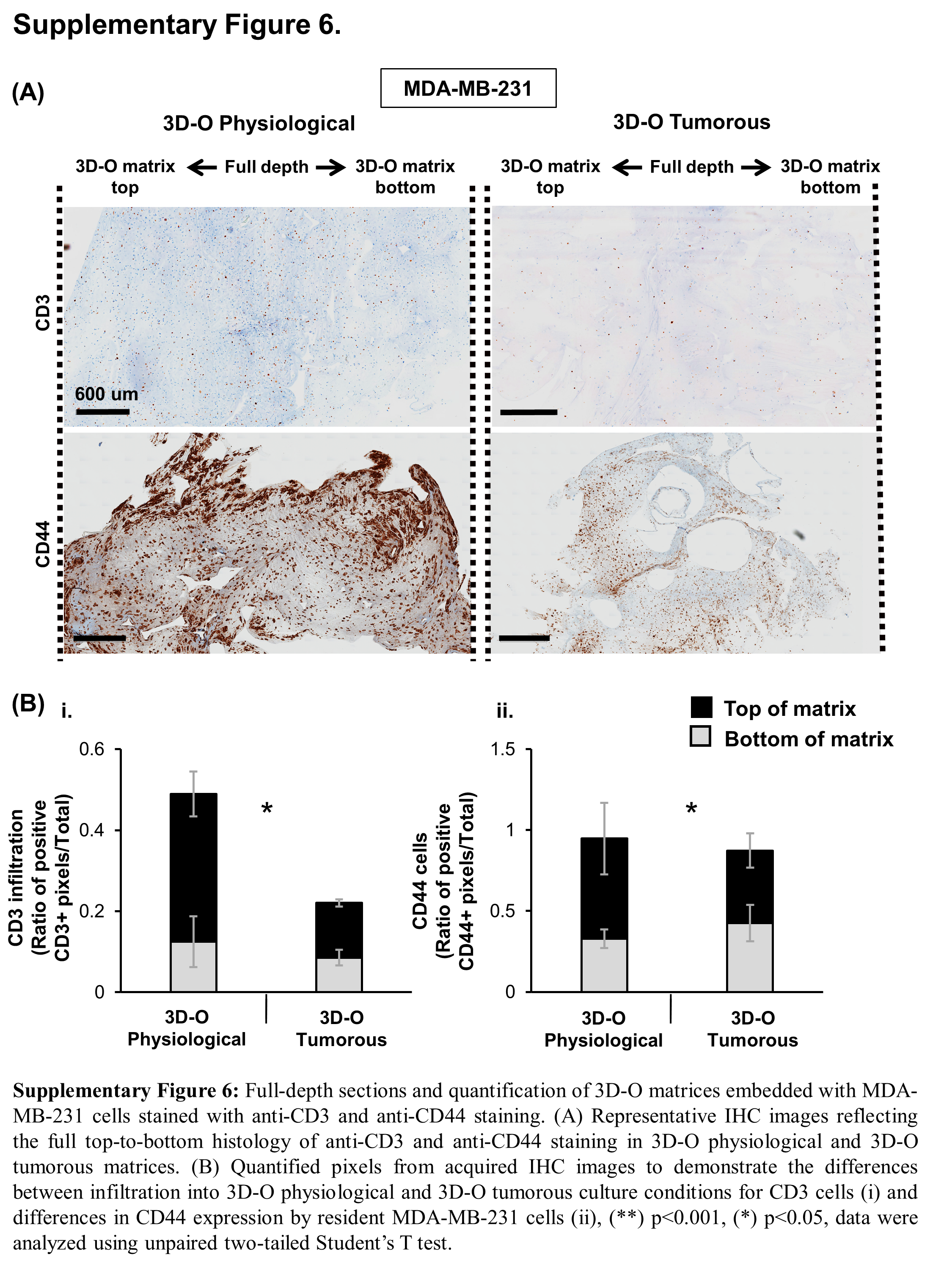

Supplement: Supplementary file 6 [file Image_6.TIF]

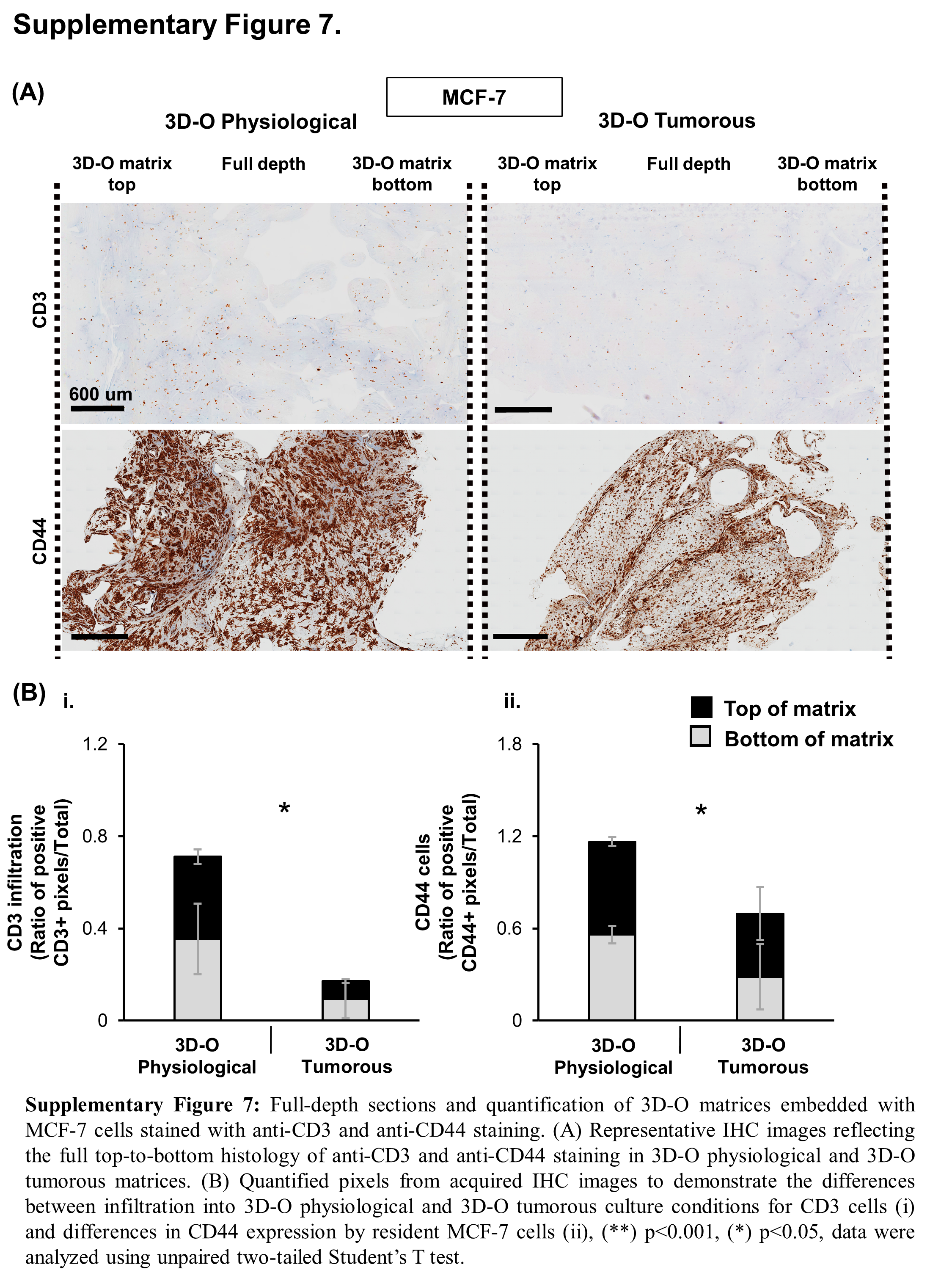

Supplement: Supplementary file 7 [file Image_7.TIF]

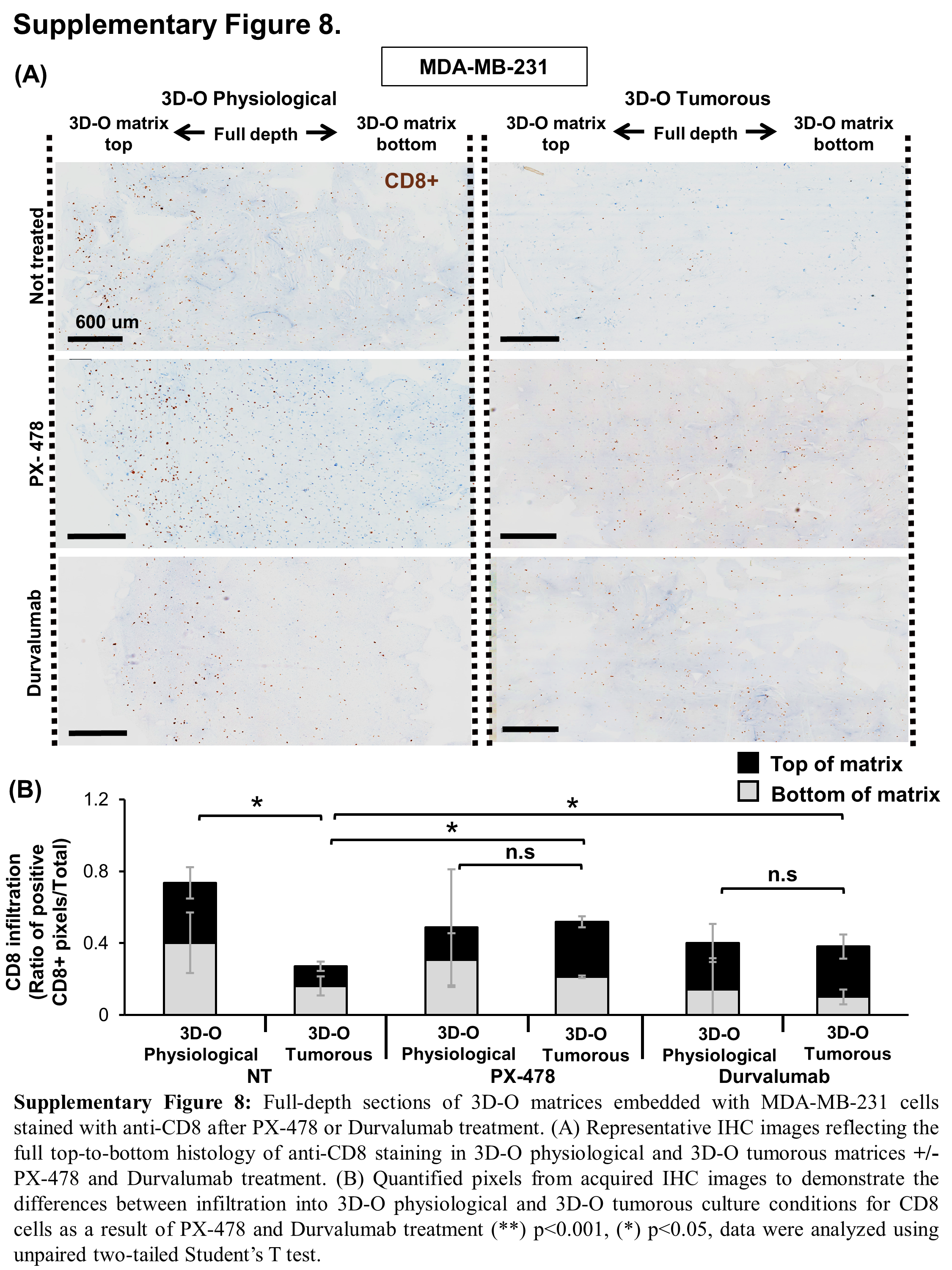

Supplement: Supplementary file 8 [file Image_8.TIF]

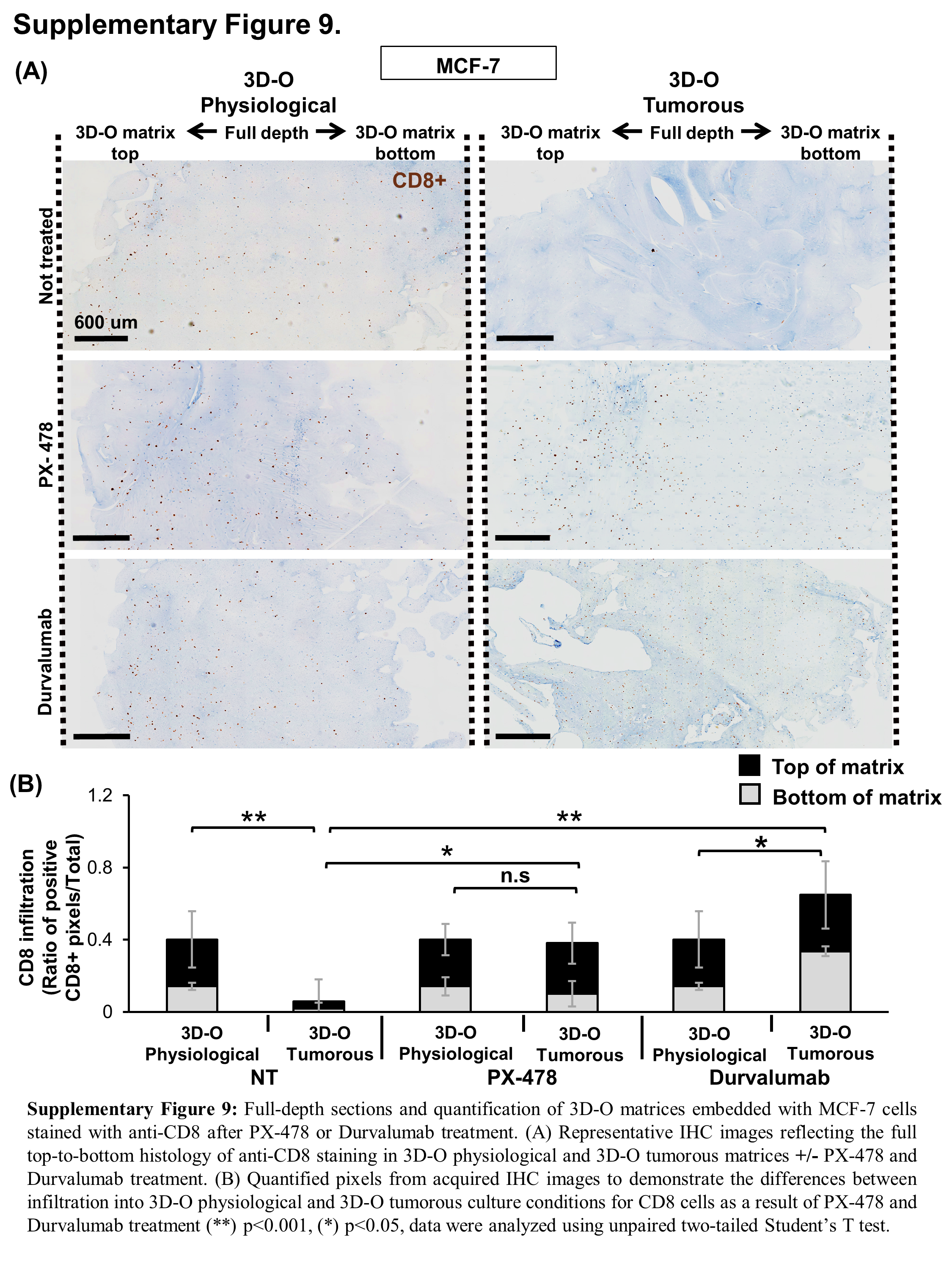

Supplement: Supplementary file 9 [file Image_9.TIF]

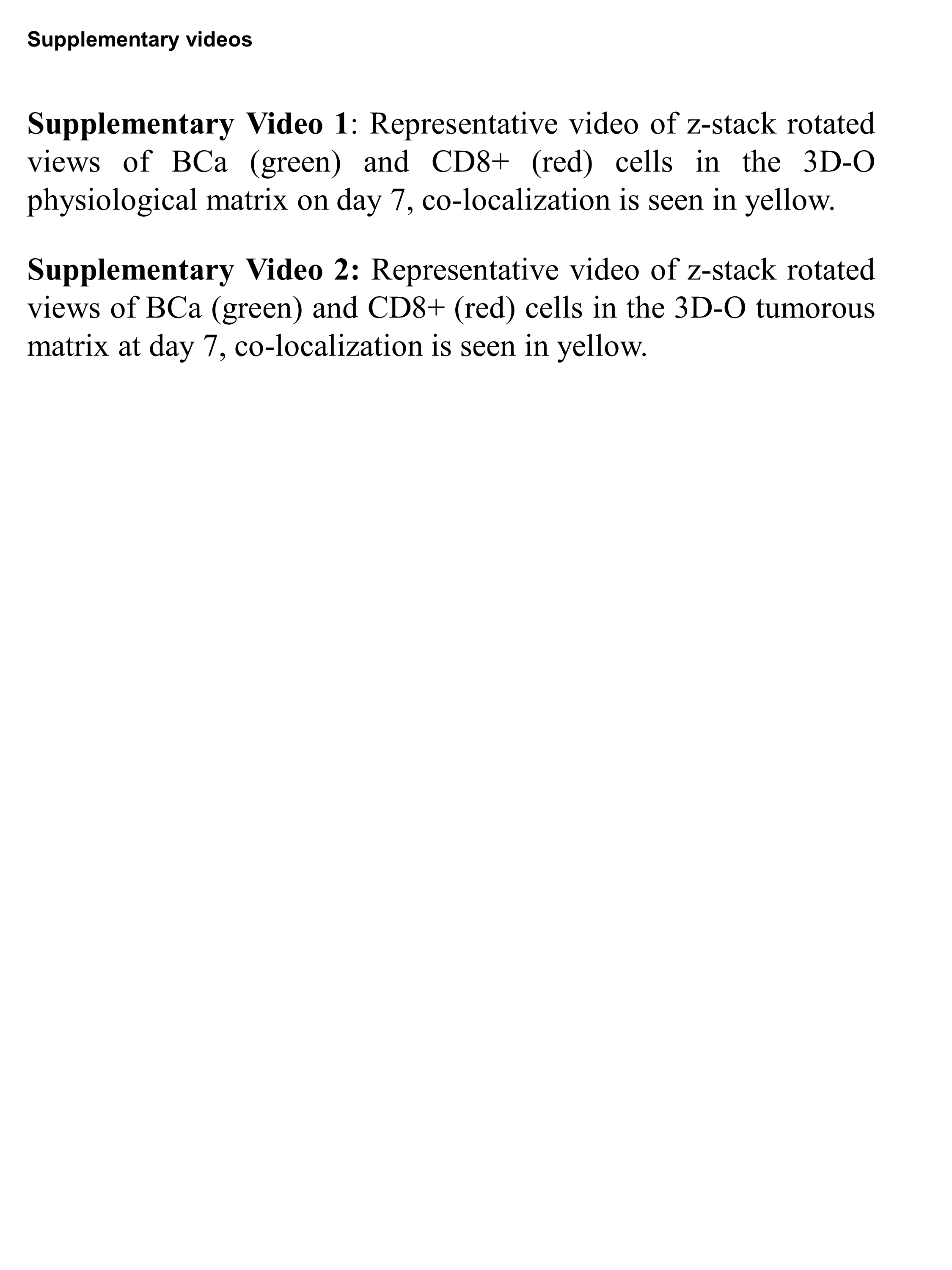

Supplement: Supplementary file 10 [file Image_10.TIF]
